# Pervasive gene flow despite strong and varied reproductive barriers in swordtails

**DOI:** 10.1038/s41559-025-02669-9

**Published:** 2025-03-26

**Authors:** Stepfanie M. Aguillon, Sophia K. Haase Cox, Quinn K. Langdon, Theresa R. Gunn, John J. Baczenas, Shreya M. Banerjee, Alexandra E. Donny, Benjamin M. Moran, Paola Fascinetto-Zago, Carla Gutiérrez-Rodríguez, Oscar Ríos-Cárdenas, Molly R. Morris, Daniel L. Powell, Molly Schumer

**Affiliations:** 1 Department of Biology, Stanford University, Stanford, CA, USA.; 2 Centro de Investigaciones Científicas de las Huastecas ‘Aguazarca’ A.C., Calnali, México.; 3 Department of Ecology and Evolutionary Biology, University of California, Los Angeles, Los Angeles, CA, USA.; 4 Gladstone Institute of Virology, Gladstone Institutes, San Francisco, CA, USA.; 5 Center for Population Biology, University of California, Davis, Davis, CA, USA.; 6 Department of Genome Sciences, University of Washington, Seattle, WA, USA.; 7 Red de Biología Evolutiva, Instituto de Ecología A.C., Xalapa, México.; 8 Department of Biological Sciences, Ohio University, Athens, OH, USA.; 9 Department of Biological Sciences, Louisiana State University, Baton Rouge, LA, USA.; 10 Freeman Hrabowski Fellow, Howard Hughes Medical Institute, Chevy Chase, MD, USA.

## Abstract

The evolution of reproductive barriers leads to the formation of new species. However, recent research has demonstrated that hybridization has been pervasive across the tree of life even in the presence of strong barriers. Using swordtail fishes (genus *Xiphophorus*), an emerging model system, we document overlapping mechanisms that act as barriers to gene flow between *Xiphophorus birchmanni* and *Xiphophorus cortezi* by combining genomic sequencing from natural hybrid populations, experimental laboratory crosses, behavioural assays, sperm measures and developmental studies. We show that assortative mating plays a role in maintaining subpopulations with distinct ancestry within natural hybrid populations. Using F_2_ hybrids we identify several genomic regions that strongly impact hybrid viability. Strikingly, two of these regions underlie genetic incompatibilities in hybrids between *X. birchmanni* and its sister species *Xiphophorus malinche*. Our results demonstrate that ancient hybridization has played a role in the origin of this shared genetic incompatibility. Moreover, ancestry mismatch at these incompatible regions has remarkably similar consequences for phenotypes and hybrid survival in *X. cortezi* × *X. birchmanni* hybrids as in *X. malinche* × *X. birchmanni* hybrids. Our findings identify varied reproductive barriers that shape genetic exchange between naturally hybridizing species and highlight the complex evolutionary outcomes of hybridization.

There are ‘endless forms’ of life on Earth, yet these diverse lineages originally trace back to a common ancestor. Understanding the mechanisms through which reproductive isolation between populations arises and leads to new species remains a foundational goal in evolutionary biology^1^. These isolating mechanisms are diverse, ranging from changes in mating preferences or reproductive timing (prezygotic barriers) to genetic changes that impact the viability or fertility of hybrids (postzygotic barriers). Despite the well-documented presence of these varied isolating mechanisms, we now know that genetic exchange through hybridization has been a pervasive evolutionary force across the tree of life^2–4^. Reconciling the prevalence of hybridization with the persistence of strong reproductive barriers between species remains a persistent puzzle.

Decades of research have led to a rich understanding of the mechanisms through which barriers to gene flow evolve^1,5,6^. Given sufficient divergence between incipient species, genomic variants will arise that differentiate lineages, and a subset of these may interact poorly when combined in hybrid genomes^7–9^. These genetic incompatibilities function as postzygotic barriers between hybridizing species and often result in inviability, reduced fertility or reduced fitness in hybrids^6^. Prezygotic behavioural barriers where individuals prefer to mate with conspecifics over heterospecifics have also been extensively documented^5^, as have preferences for different environmental factors, which can lead to similar dynamics^10^. Initially, different isolating mechanisms may work independently to incompletely limit gene flow between incipient species, but over time they may interact to strengthen or couple and form more complete barriers to genetic exchange^11–13^. In concert, multiple mechanisms are predicted to more completely reduce genetic exchange between diverging lineages^13–16^.

The increasing availability of genomic data has exposed how this classic view of the evolution of reproductive isolation is discordant with patterns observed in many species. For example, in groups such as *Drosophila*^17,18^ and *Heliconius*^19–21^, both historical and contemporary genetic exchanges are common between lineages, despite the presence of multiple, strong isolating barriers. This raises fundamental questions about how isolating barriers interact—and potentially evolve—in the face of repeated and ongoing gene flow between species over evolutionary time^3,22^. While the effects of hybridization on the movement of alleles underlying adaptive traits have long been recognized^3,23^, the broader consequences for reproductive isolation as a result of the exchange of genetic incompatibilities have been less thoroughly investigated (but see refs. 24–27). Historically, the field has assumed that without extremely strong reproductive isolation hybridization will erase behavioural preferences, environmental adaptations or genetic incompatibilities that distinguish hybridizing lineages^1,28^. However, the increased appreciation of the complexity of hybridization on a phylogenetic scale—with many species simultaneously exchanging genes^29–32^—complicates this expectation. Instead, introgression of genes that impact reproductive isolation between two species could have secondary consequences on reproductive isolation when hybridization occurs with additional species where these barriers did not originally evolve. Such dynamics would have important implications for our understanding of how reproductive barriers evolve and persist in nature.

We leverage naturally hybridizing species of swordtails (*Xiphophorus birchmanni* and *Xiphophorus cortezi*), live-bearing freshwater fish native to eastern México, to explore their complex reproductive barriers and how hybridization interacts with these barriers in nature. Past work in *Xiphophorus* has explored the role of a variety of isolating mechanisms independently: including, genetic incompatibilities^33–35^, genomic architecture of important traits^32,36^, mate preferences^37–39^ and ecological differences^40^. First, we combine extensive genomic sampling of a newly identified hybrid population with mate choice assays and paired mother/embryo sequencing to explore the role of assortative mating in the wild. Next, using artificial crosses in the laboratory, we characterize the viability of hybrids and compare sperm morphology and motility between parental species and hybrids. Finally, we leverage genome-wide data from second-generation laboratory-generated hybrids to characterize genetic incompatibilities and their phenotypic consequences. Despite ongoing gene flow between *X. cortezi* and *X. birchmanni*, we find evidence for both prezygotic and postzygotic isolating mechanisms working in concert to form strong but incomplete reproductive barriers. Moreover, we explicitly test the role of loci that have introgressed into *X. cortezi* from a third species on reproductive isolation between *X. cortezi* and *X. birchmanni*. Results of these experiments provide one of the most comprehensive descriptions of how introgression can spread genetic incompatibilities to additional species pairs. This finding has profound implications for our understanding of how isolating barriers evolve in the face of gene flow.

## Results

### Genomic ancestry in a new hybrid population

**Extended Data Fig. 1 |:**
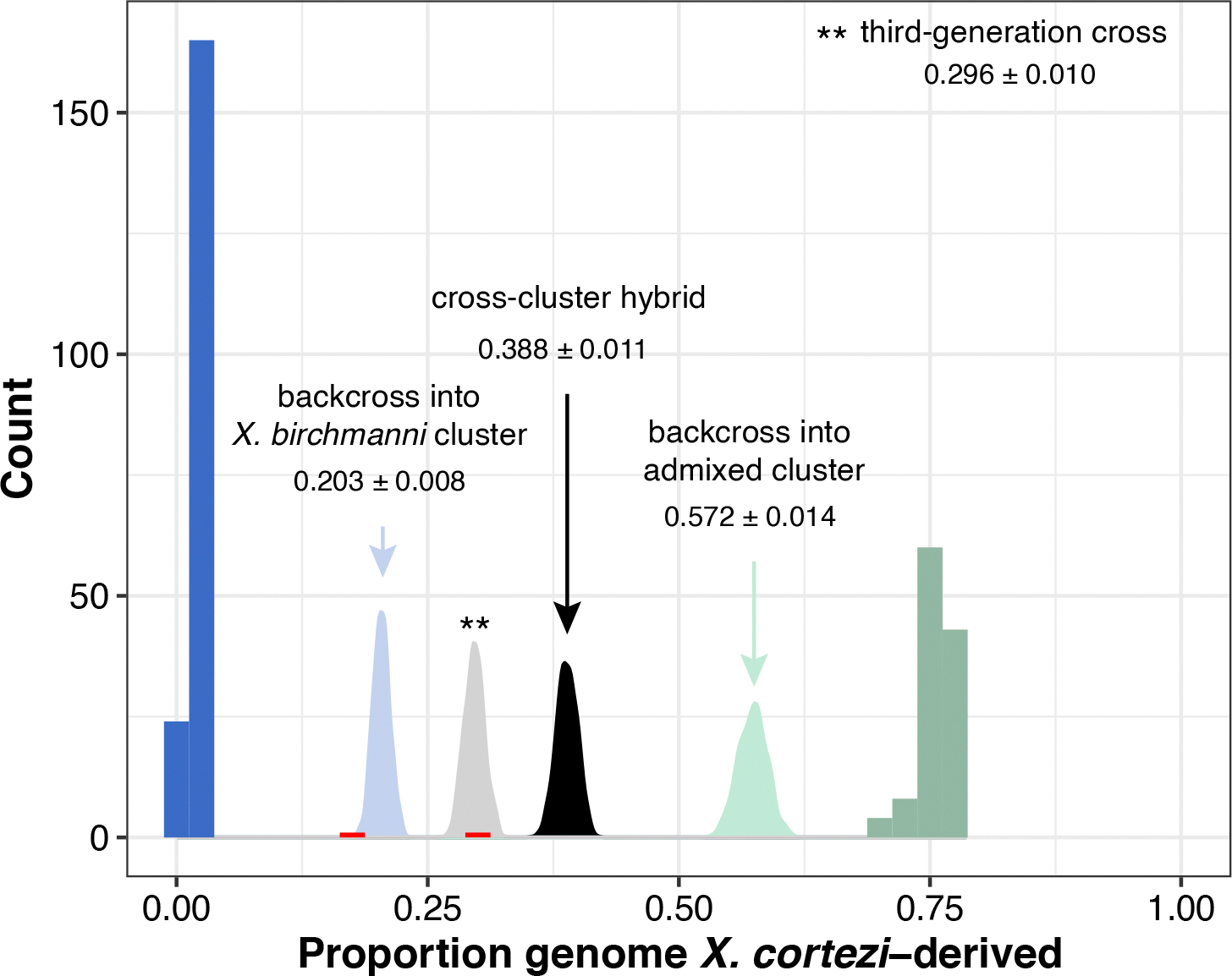
Simulations of cross-cluster hybridization events. Distribution of genome-wide ancestry in Chapulhuacanito showing observed data as a histogram (as in Fig. 1b) with the *X. birchmanni* cluster in blue, admixed cluster in light green, and intermediate ancestry individuals highlighted in red. We conducted simulations of recent generation cross-cluster mating events in R (see Supplementary Discussion 1 for details). We found that one of the intermediate individuals falls within the simulated ancestry distribution of backcross offspring between a first-generation cross-cluster hybrid and a *X. birchmanni* cluster individual (light blue distribution). Observed ancestry tracts of this individual (Supplementary Fig. 1c) also support this conclusion. The other intermediate individual fell within the simulated ancestry distribution of a third-generation cross (light gray) between a cross-cluster hybrid and a *X. birchmanni*-backcrossed individual (ancestry tracts in Supplementary Fig. 1b).

**Fig. 1 |:**
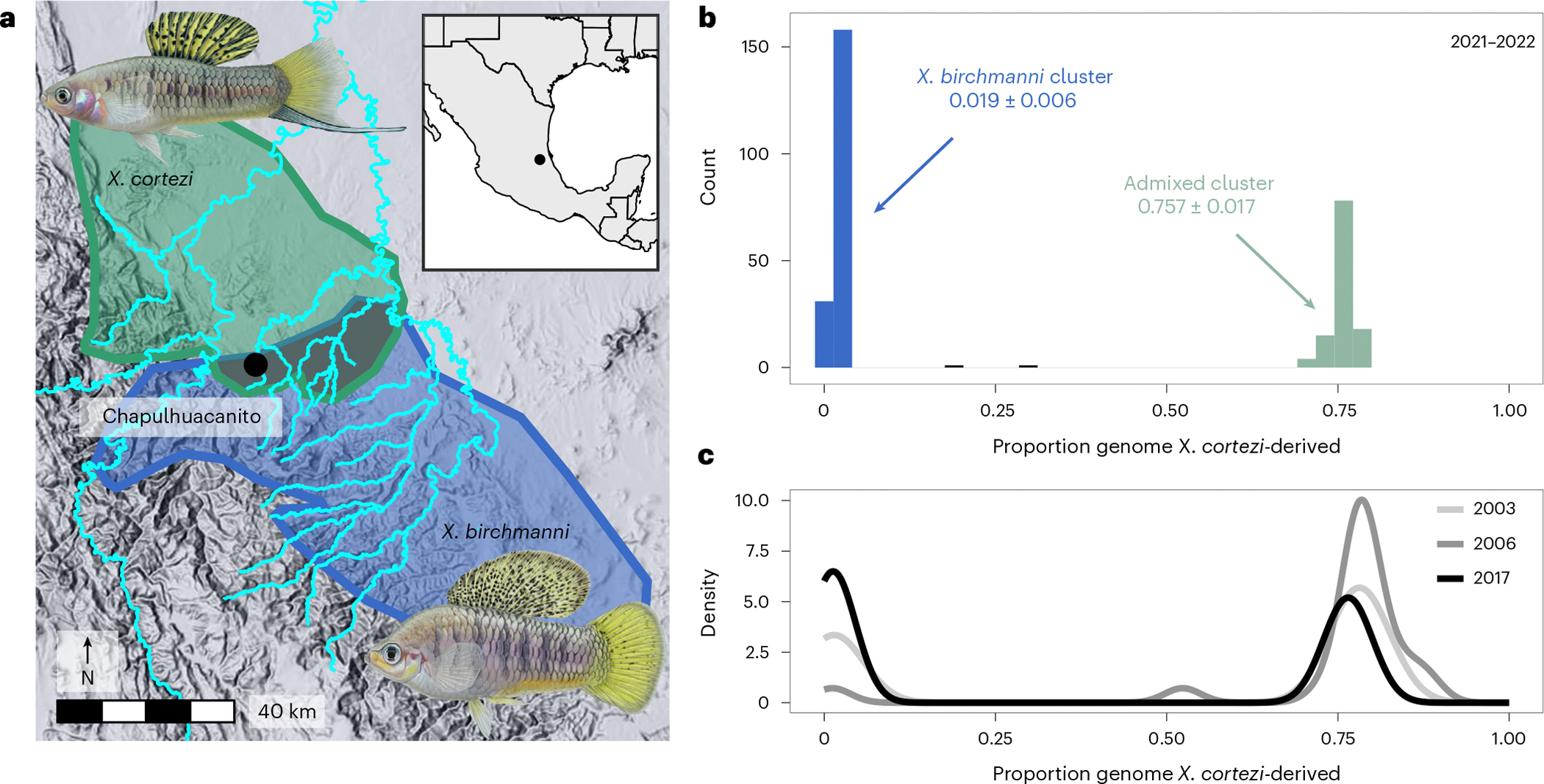
Distribution of genome-wide ancestry in a newly described hybridizing population between *X. cortezi* and *X. birchmanni.* **a**, The Chapulhuacanito population is located along a tributary of the Río San Pedro where the *X. cortezi* (green) and *X. birchmanni* (blue) ranges overlap. Illustrations of males of each species by Dorian Noel. **b**, This population displays strong bimodality in genome-wide ancestry (one-sided Hartigan’s dip statistic for unimodality: *n* = 306, *D* = 0.166, *P* < 2.2 × 10^−16^) with individuals primarily falling into two ancestry the genome (0.019 ± 0.006, blue), while ~38% are admixed between the two species (0.757 ± 0.017, light green). **c**, The strong bimodality present in contemporary samples has been present in this population for at least the past 19 years or ~40 generations (one-sided Hartigan’s dip statistic for unimodality: *n* = 73, *D* = 0.180, *P* < 2.2 × 10^−16^ for historical samples).

We applied whole-genome sequencing and local ancestry inference^41,42^ to characterize the genomic ancestry of 306 adults sampled in 2021–2022 from Chapulhuacanito, a recently identified hybrid population between *X. birchmanni* and *X. cortezi* (Fig. 1a). Using posterior probabilities of ancestry at ~1 million informative sites across the genome, we calculated the proportion of the genome derived from each parental species. We found a strong bimodal distribution of ancestry proportions among sampled individuals (Fig. 1b; one-sided Hartigan’s dip statistic for unimodality, *D* = 0.166, *P* < 2.2 × 10^−16^). Adults typically fell into one of two ancestry clusters: ~62% belonged to a nearly pure *X. birchmanni* cluster deriving only 1.9 ± 0.6% (mean ± s.d.) of their genome from *X. cortezi*, whereas ~38% belonged to an admixed cluster deriving 75.7 ± 1.7% of their genome from *X. cortezi* (Fig. 1b). This bimodal distribution of ancestry is strikingly similar to that found in an independently formed hybrid population between *X. birchmanni* and *X. cortezi* in the Río Santa Cruz^42^, highlighting repeatable evolutionary outcomes in these replicated instances of natural hybridization. Despite the strong bimodal population structure, we also identified two individuals with intermediate ancestry proportions (Fig. 1b). Simulations of mating events (Supplementary Discussion 1 and Extended Data Fig. 1) and patterns of local ancestry (Supplementary Fig. 1) confirm that both individuals are the product of recent-generation mating events between the two ancestry clusters.

To understand whether the strong population structure we observed at Chapulhuacanito was stable over time, we leveraged genomic data from a companion study^43^ that included historical samples from 2003 (*n* = 11), 2006 (*n* = 21) and 2017 (*n* = 41). We found a similar bimodal distribution of ancestry in these historical collections (Fig. 1c and Supplementary Fig. 2; one-sided Hartigan’s dip statistic for unimodality across years, *D* = 0.180, *P* < 2.2 × 10^−16^; see Supplementary Table 1 for analyses separated by year), demonstrating that ancestry structure in this population has been stable for at least ~40 generations (Supplementary Table 2).

### Assortative mating is present in the wild

**Extended Data Fig. 2 |:**
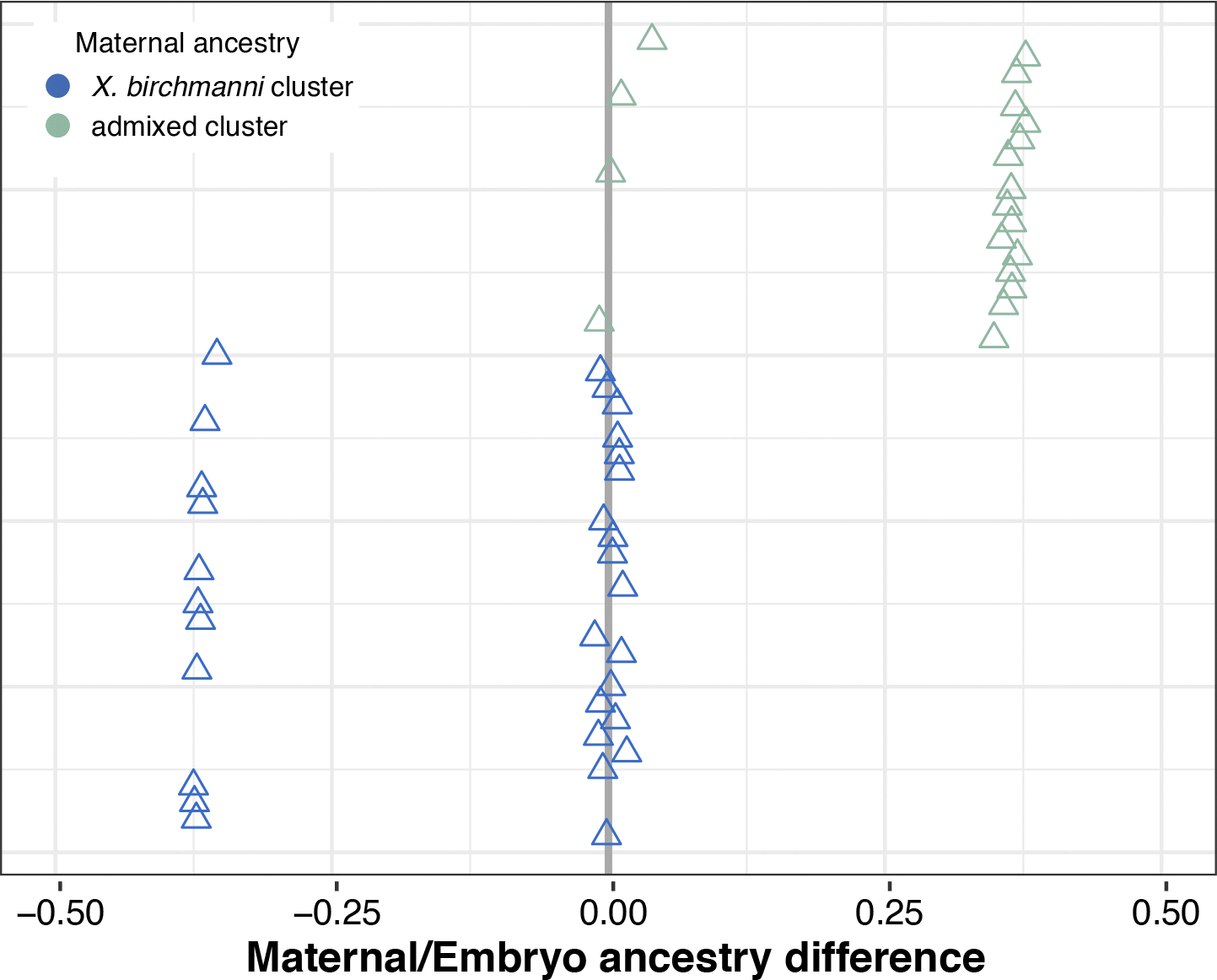
Simulations of random mating predict many cross-cluster mating events in the Chapulhuacanito population. This can be observed by the many points where the difference in genome-wide ancestry between simulated females and their embryos is far from the zero-line. This contrasts strongly from the observed pattern of complete assortative mating shown in Fig. 2a. Points are ordered along the y-axis by increasing maternal *X. cortezi*-derived genomic ancestry. The zero-line indicates a difference between maternal/embryo ancestry of zero.

**Fig. 2 |:**
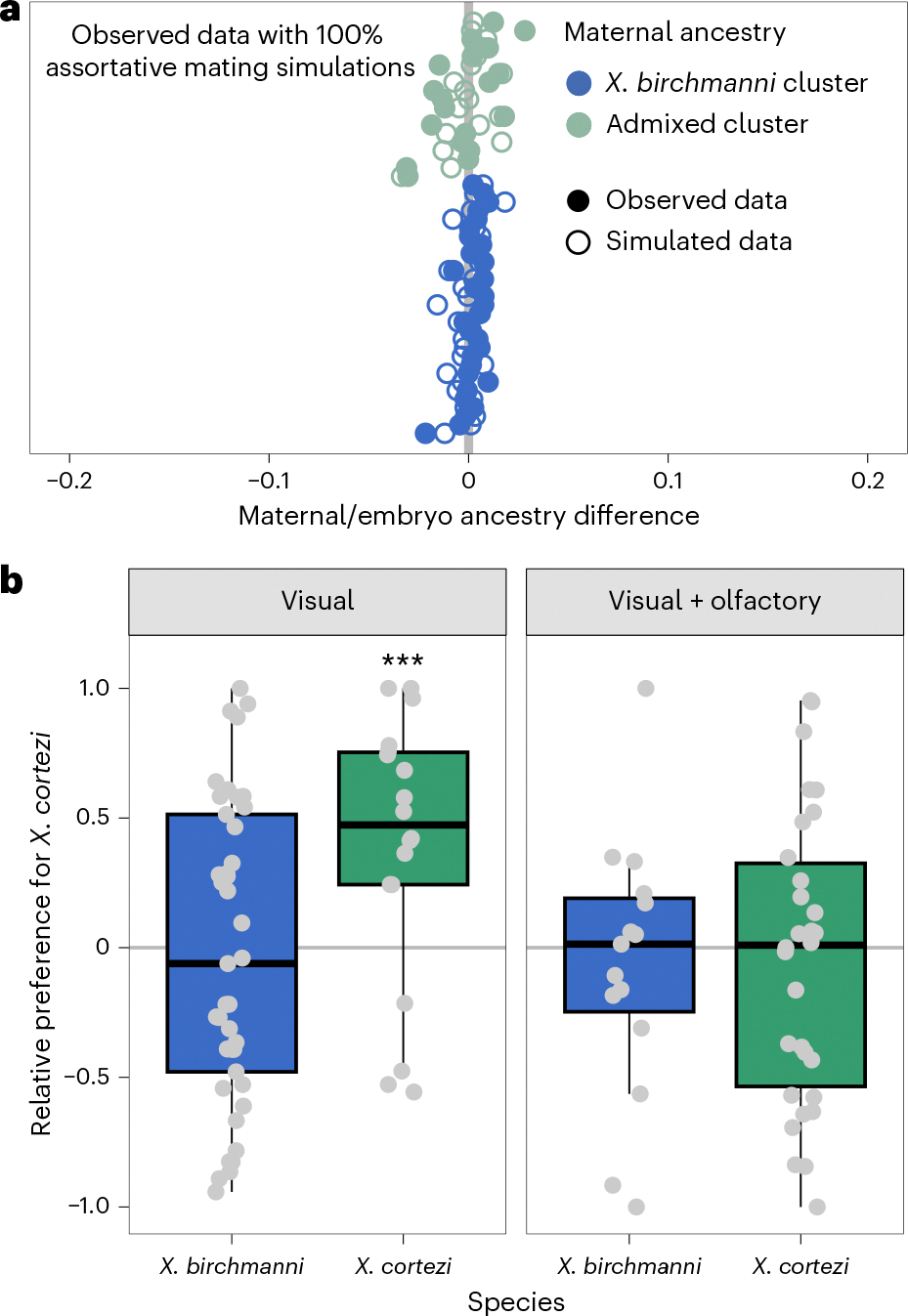
Assortative mating by ancestry in the wild is strong, but not explained by in-laboratory female preference trials. **a**, Paired mother/embryo sequencing provides evidence for assortative mating in Chapulhuacanito. The differences in observed genome-wide ancestry between females and their embryos (closed circles) are tightly aligned with the zero-line, indicating that females from both ancestry clusters mated exclusively with males from their own cluster. Simulations of complete assortative mating by ancestry (open circles) most closely match our observations. See Extended Data Fig. 2 for a simulation of random mating. Points are ordered along the *y* axis by increasing maternal *X. cortezi*-derived genomic ancestry. The zero-line indicates a difference between maternal and embryo ancestry of zero. **b**, Female mate preferences in allopatric individuals of the two hybridizing species are complex. *X. birchmanni* females (blue boxplots) lack preferences for either conspecific or heterospecific males in both visual (one-sided Wilcoxon signed-rank test: *n* = 41, *P* = 0.4288) and visual with olfactory (*n* = 37, *P* = 0.5932) trials. By contrast, *X. cortezi* females (green boxplots) showed strong preferences for conspecific males in visual trials (*n* = 18, ***P = 0.0058), but lacked preferences in trials where both visual and olfactory cues were included (*n* = 30, *P* = 0.6289). Relative preference for *X. cortezi* is calculated as the difference between time spent with the *X. cortezi* cue and time spent with the *X. birchmanni* cue, divided by the total time spent with either. Positive values indicate preference for *X. cortezi* males, while negative values indicate preference for *X. birchmanni* males. The bold centre line is the median, coloured box limits are the interquartile range (IQR) and the whiskers show 1.5× IQR.

The strongly bimodal ancestry distribution in Chapulhuacanito hints that assortative mating may exist between the *X. birchmanni* and admixed clusters through mate choice, sperm use or differences in fertilization success (see Supplementary Discussion 2 for alternatives). To directly test for evidence of assortative mating, we leveraged the unique biology of these live-bearing fish: we performed whole-genome sequencing on pregnant females collected from the wild (*n* = 49) and at least two of their developing embryos (*n* = 101). We compared the difference between the genome-wide ancestry of the mother and her embryos to infer the father’s ancestry. Matings within the same cluster are predicted to result in small differences in ancestry between the female and her embryos, while cross-cluster matings result in larger differences (from simulations, on average 36.7% ± 0.72% in this population; Extended Data Fig. 2). Across all mother/embryo pairs, we found no evidence for cross-cluster mating (Fig. 2a and Supplementary Fig. 3), allowing us to definitively reject a model of random mating in this population. In fact, by parameterizing simulations with observed ancestry data (Supplementary Discussion 3), we found that complete assortative mating by ancestry provides the best fit to our data (Fig. 2a and Supplementary Fig. 4). Because we identified a few intermediate individuals in our larger dataset (see previous section), we know assortative mating by ancestry is not always complete. However, these results are consistent with power limitations expected from the sample size of our mother/embryo dataset (Supplementary Fig. 5). Although embryonic lethality of cross-cluster embryos could also explain this pattern, other results demonstrate that we can effectively sample inviable embryos, suggesting that this is not a strong driver of our results (Supplementary Discussion 2).

### Female behavioural trials do not explain assortative mating

**Extended Data Fig. 3 |:**
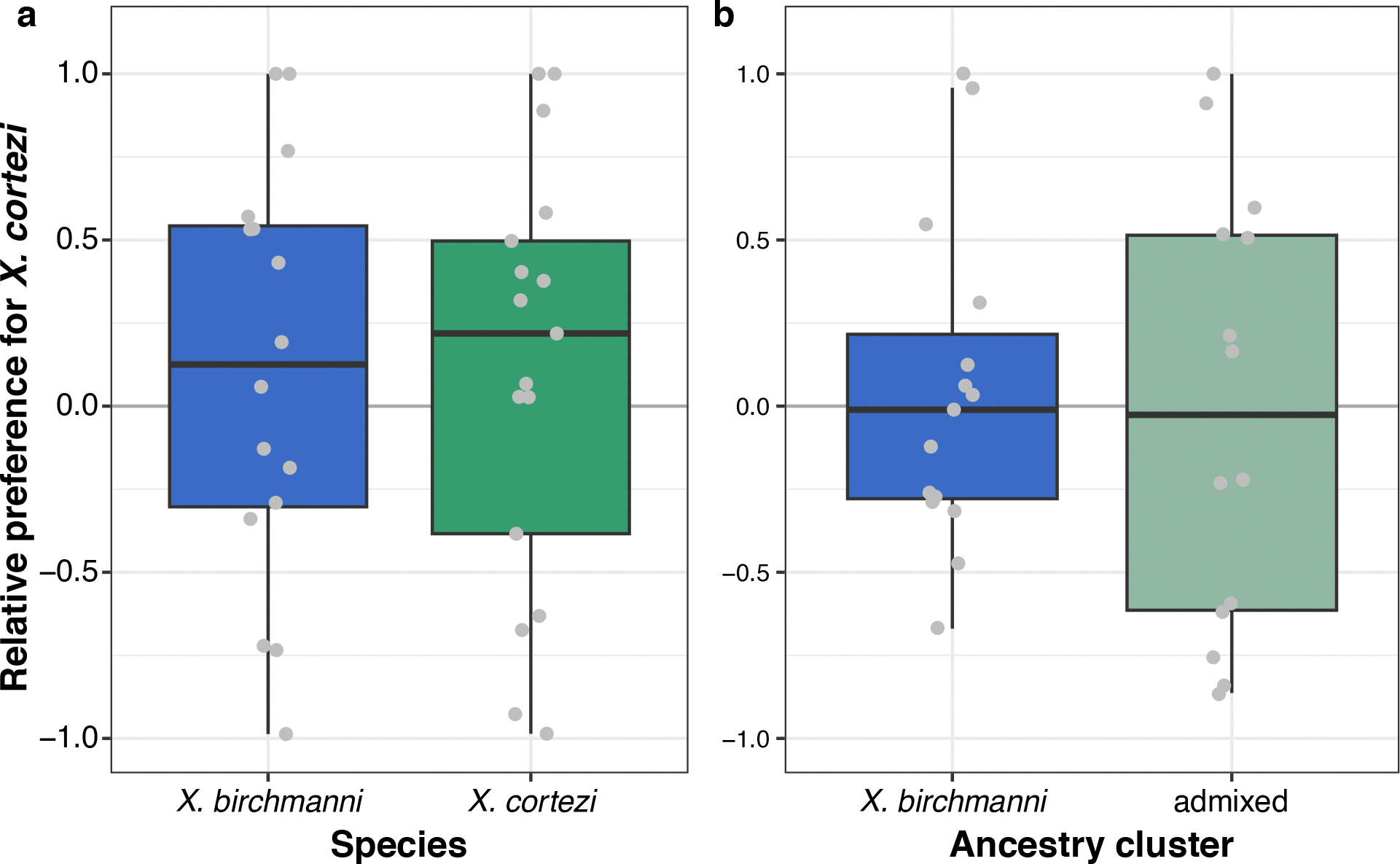
Female preference trials using isolated male pheromone cues. Results of mate preference trials using isolated male pheromone cues to test preferences of *X. birchmanni* versus *X. cortezi* pheromones in (**a**) allopatric *X. birchmanni* and *X. cortezi* females and (**b**) females from the two ancestry clusters in the hybrid population Chapulhuacanito. Experimental details can be found in Supplementary Discussion 4. *X. birchmanni* females (blue boxplots) lack preferences for either con- or hetero-specific males in both allopatric populations (**a**, one-sided Wilcoxon signed rank test: *n* = 16, *P* = 0.7886) and in the Chapulhuacanito population (**b**, one-sided Wilcoxon signed rank test: *n* = 15, *P* = 0.511). Similarly, allopatric *X. cortezi* females (**a**, *n* = 17, *P* = 0.2105) and females from the admixed ancestry cluster in Chapulhuacanito (**b**, *n* = 14, *P* = 0.5961) lack preferences for either con- or hetero-specific males (green boxplots). Relative preference for *X. cortezi* is calculated as the difference between time spent with the *X. cortezi* cue and time spent with the *X. birchmanni* cue, divided by the total time spent with either. Positive values indicate preference for *X. cortezi* males, while negative values indicate preference for *X. birchmanni* males. The bold center line is the median, colored box limits are the interquartile range (IQR), and the whiskers show 1.5 × IQR.

**Extended Data Fig. 4 |:**
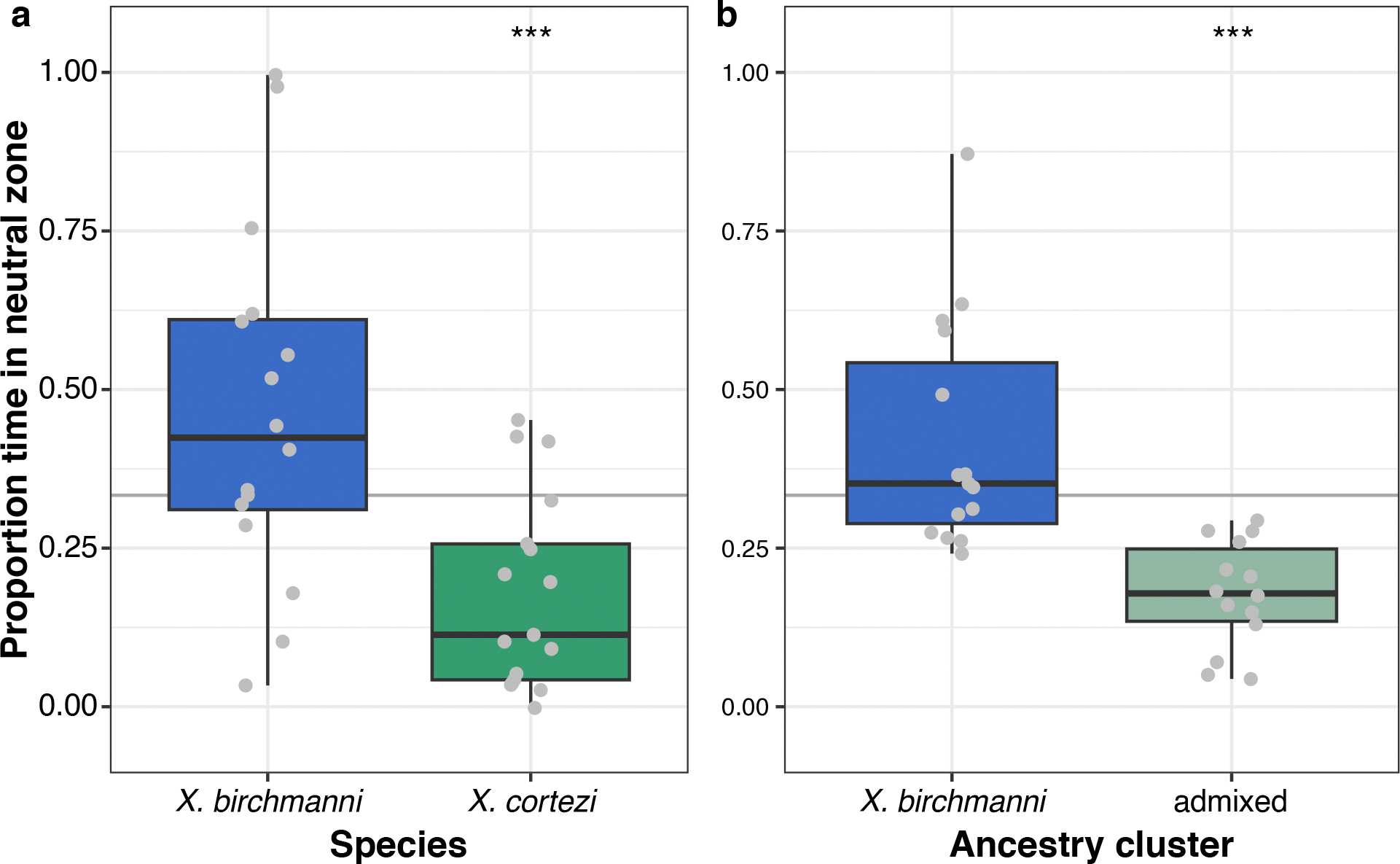
Behavioral differences observed during isolated male pheromone trials. Difference in behavior of females during mate preference trials of (**a**) allopatric *X. birchmanni* and *X. cortezi* females and (**b***)* females from the two ancestry clusters in Chapulhuacanito. Experimental details can be found in Supplementary Discussion 4. Allopatric *X. cortezi* females (**a**, one-sided Wilcoxon signed-rank test: *n* = 17, *P* = 0.0017***) and females from the admixed ancestry cluster in Chapulhuacanito (**b**, one-sided Wilcoxon signed rank test: *n* = 14, *P* = 0.0001***) spent significantly less time in the neutral zone than expected by random chance (green boxplots). Whereas *X. birchmanni* females (blue boxplots) did not differ from random chance in either allopatric (**a**, *n* = 16, *P* = 0.1167) or Chapulhuacanito (**b**, *n* = 15, *P* = 0.3028) populations. In both the allopatric comparison (**a**, *n* = 33, *P* = 0.0016) and the Chapulhuacanito comparison (**b**, *n* = 29, *P* = 9.6 × 10^−6^), *X. birchmanni* females spent significantly less time in the neutral zone than pure *X. cortezi* or admixed hybrid females, respectively. Because trial lanes are divided into 3 equal sections, we define random chance as 1/3 of the total trial time (shown here with a gray horizontal line). The bold center line is the median, colored box limits are the interquartile range (IQR), and the whiskers show 1.5 × IQR.

To investigate behavioural mechanisms that might be linked to assortative mating in these species, we collected *X. birchmanni* and *X. cortezi* individuals from allopatric populations to test the presence and strength of conspecific mating preferences. Female preferences for conspecific male visual and olfactory cues are common across *Xiphophorus*^39,44,45^ and are thought to be important in maintaining isolation between species^46^. However, mating preferences have not been studied in the context of hybridization between *X. birchmanni* and *X. cortezi*. Despite clear genetic evidence of assortative mating by ancestry, we found complex results from behavioural trials (Fig. 2b). Surprisingly, *X. birchmanni* females did not demonstrate preferences for conspecific males in either visual (one-sided Wilcoxon signed-rank test, *n* = 41, *P* = 0.4288) or visual with olfactory (*n* = 37, *P* = 0.5932) trials. By contrast, *X. cortezi* females strongly preferred conspecific males in visual trials (*n* = 18, *P* = 0.0058), but not when olfactory cues were included (*n* = 30, *P* = 0.6289). On the basis of data from other *Xiphophorus* species, we hypothesized that these species may differ in released pheromones and associated preferences^47,48^. However, in separate experiments we did not recover mating preferences using isolated male pheromones in females from allopatric populations (*n* = 16 *X. birchmanni*, *n* = 17 *X. cortezi*) or from either ancestry cluster (*n* = 15 *X. birchmanni*, *n* = 14 admixed cluster) in the sympatric Chapulhuacanito population (Extended Data Figs. 3 and 4 and Supplementary Discussion 4). Taken together, our behavioural experiments do not clearly explain the assortative mating patterns observed in Chapulhuacanito. Instead, they suggest that barriers to gene flow between these two species involve more than just prezygotic mechanisms. However, we caution against overinterpretation of behavioural results given high individual variability and low power in these trials and the fact that studies were based on both allopatric and sympatric populations (Supplementary Discussion 5).

### Dysfunction in artificial crosses and differences in sperm

To begin to characterize postzygotic mechanisms that may function as barriers to gene flow between these species, we produced artificial F_1_ hybrids between *X. cortezi* and *X. birchmanni* in laboratory mesocosms (Methods). Strikingly, we found dysfunction in both directions of the cross, although of differing types (Supplementary Table 3). The cross with *X. cortezi* females and *X. birchmanni* males produced F_1_s with a heavily skewed sex ratio (15.6% males; two-sided exact binomial test: *P* = 0.0001), while the alternate cross was largely unsuccessful (although artificial insemination experiments showed fertilization could occur, Supplementary Fig. 6). See Supplementary Discussion 6 for further details.

**Extended Data Fig. 5 |:**
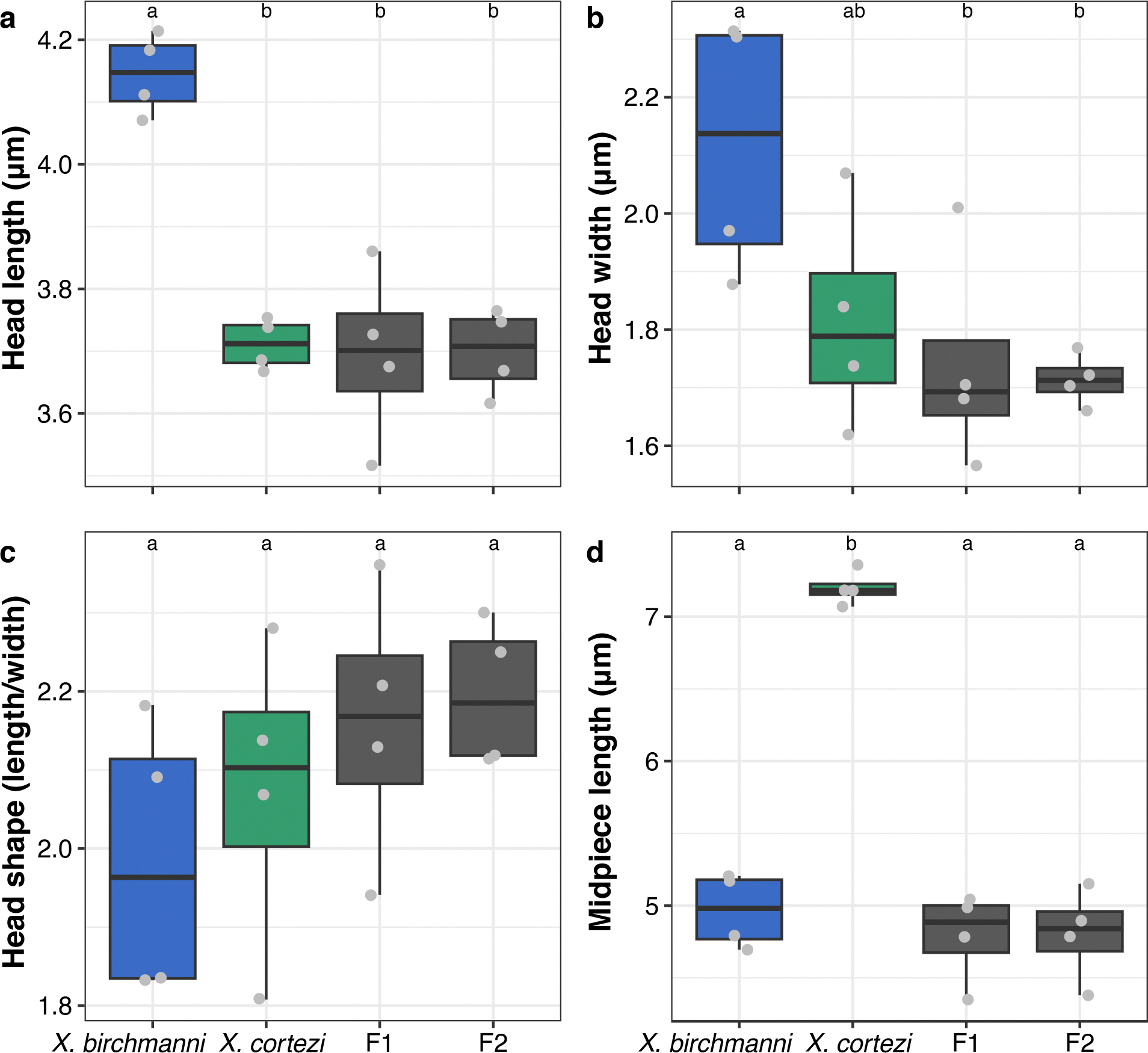
Characterization of sperm morphology. Differences in sperm morphology between *X. birchmanni* (blue), *X. cortezi* (green), and their F_1_ and F_2_ hybrids (gray) for (**a**) sperm head length, (**b**) sperm head width, (**c**) sperm head shape, and (**d**) sperm midpiece length. Points represent average values from 10 sperm from each male, with *n* = 4 males sampled per group. Sperm head shape is measured as the proportion of head length to head width. *X. birchmanni* sperm had significantly longer heads than all other groups and significantly wider heads than all other groups except *X. cortezi. X. cortezi* sperm had significantly longer midpieces than all other groups. Head shape did not significantly differ between groups. See Supplementary Table 4 for associated statistical analyses and Supplementary Discussion 6 for detailed methods and results. Letters above boxplots indicate statistical differences using two-sided Wilcoxon rank sum tests. The bold center line is the median, colored box limits are the interquartile range (IQR), and the whiskers show 1.5 × IQR.

**Extended Data Fig. 6 |:**
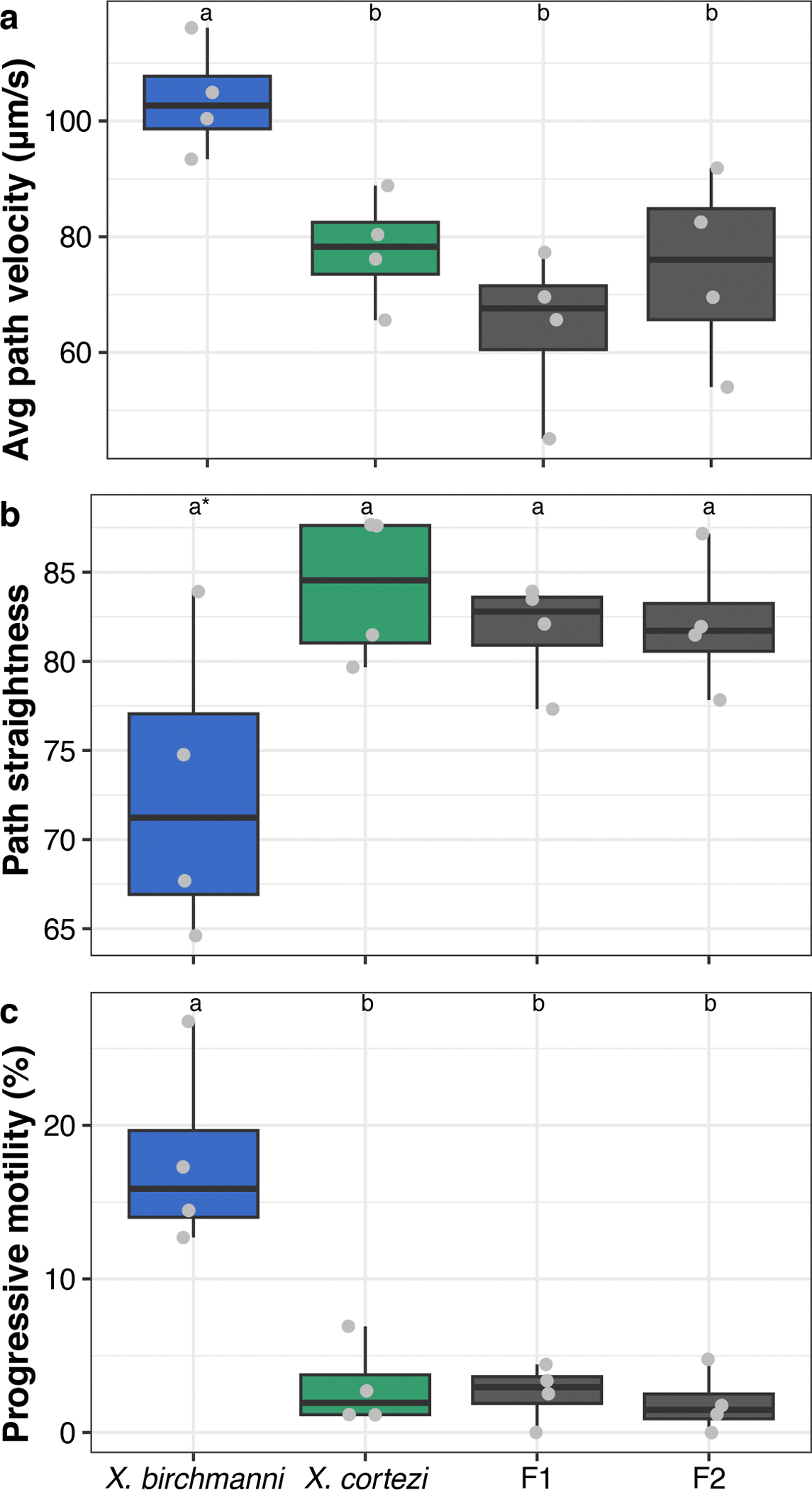
Characterization of sperm motility. Differences in sperm motility between *X. birchmanni* (blue), *X. cortezi* (green), and their F_1_ and F_2_ hybrids (gray). Points represent average values from 10 sperm from each male, with *n* = 4 males sampled per group. **a**, Average-path velocity (VAP) was greater in *X. birchmanni* than all other groups. **b**, *X. cortezi* sperm swam marginally straighter paths (STR) than *X. birchmanni*, but not hybrid sperm. **c**, *X. birchmanni* sperm had greater progressive motility than all other groups. See Supplementary Table 4 for associated statistical analyses and Supplementary Discussion 6 for detailed methods and results. Letters above boxplots indicate statistical differences using two-sided Wilcoxon rank sum tests. The bold center line is the median, colored box limits are the interquartile range (IQR), and the whiskers show 1.5 × IQR.

Some barriers to gene flow act after mating occurs but before zygotes are formed^49^. We assessed sperm morphology and motility in four individuals each of *X. birchmanni*, *X. cortezi* and their F_1_ and F_2_ hybrids. We found evidence of species-level differences and recombinant hybrid phenotypes in sperm morphology (Supplementary Table 4 and Extended Data Fig. 5), as well as differences in sperm motility between groups (Extended Data Fig. 6). Given the large differences detected between the two species, these results hint at the possibility that postmating prezygotic mechanisms (through sperm storage or cryptic female choice^50^) could impact fertilization success and lead to a higher frequency of conspecific fertilizations. See Supplementary Discussion 7 for further details.

### An introgressed incompatibility strongly influences hybrids

**Extended Data Fig. 7 |:**
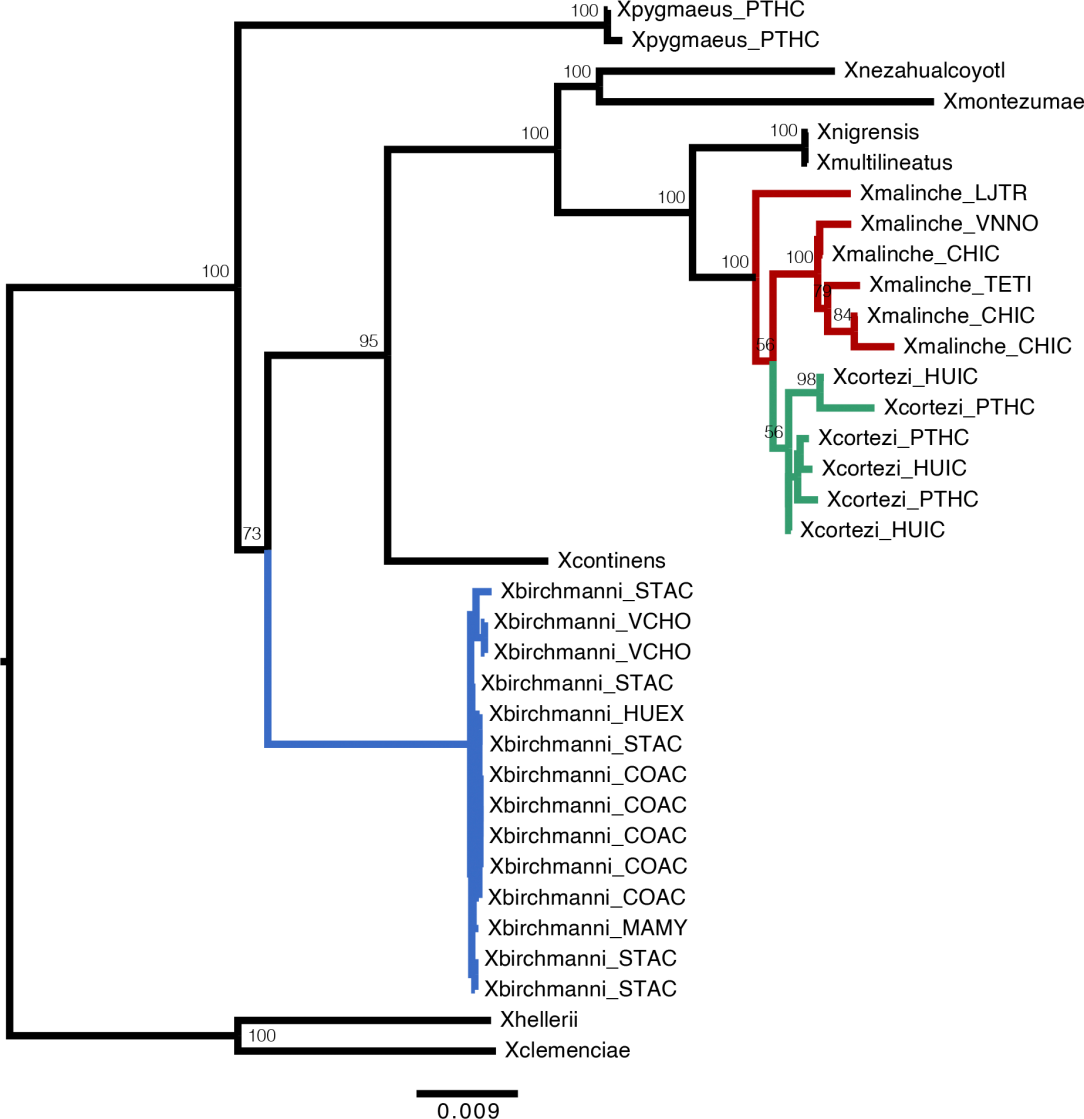
Mitochondrial phylogeny of a diverse sample of *Xiphophorus.* Mitochondrial phylogeny of a geographically diverse sample of *X. birchmanni* (blue), *X. malinche* (red), *X. cortezi* (green), and their close relatives produced using RAxML. *X. cortezi* mitochondrial diversity is clustered within the *X. malinche* mitochondrial clade and is strongly separated from *X. birchmanni*. The *X. birchmanni* clade includes samples allopatric from both *X. malinche* and *X. cortezi*, as well as samples from sympatric *X. birchmanni* from the hybrid population in the Río Santa Cruz. Node support is shown for the major clades and a scale bar for branch length is included at the bottom.

**Fig. 3 |:**
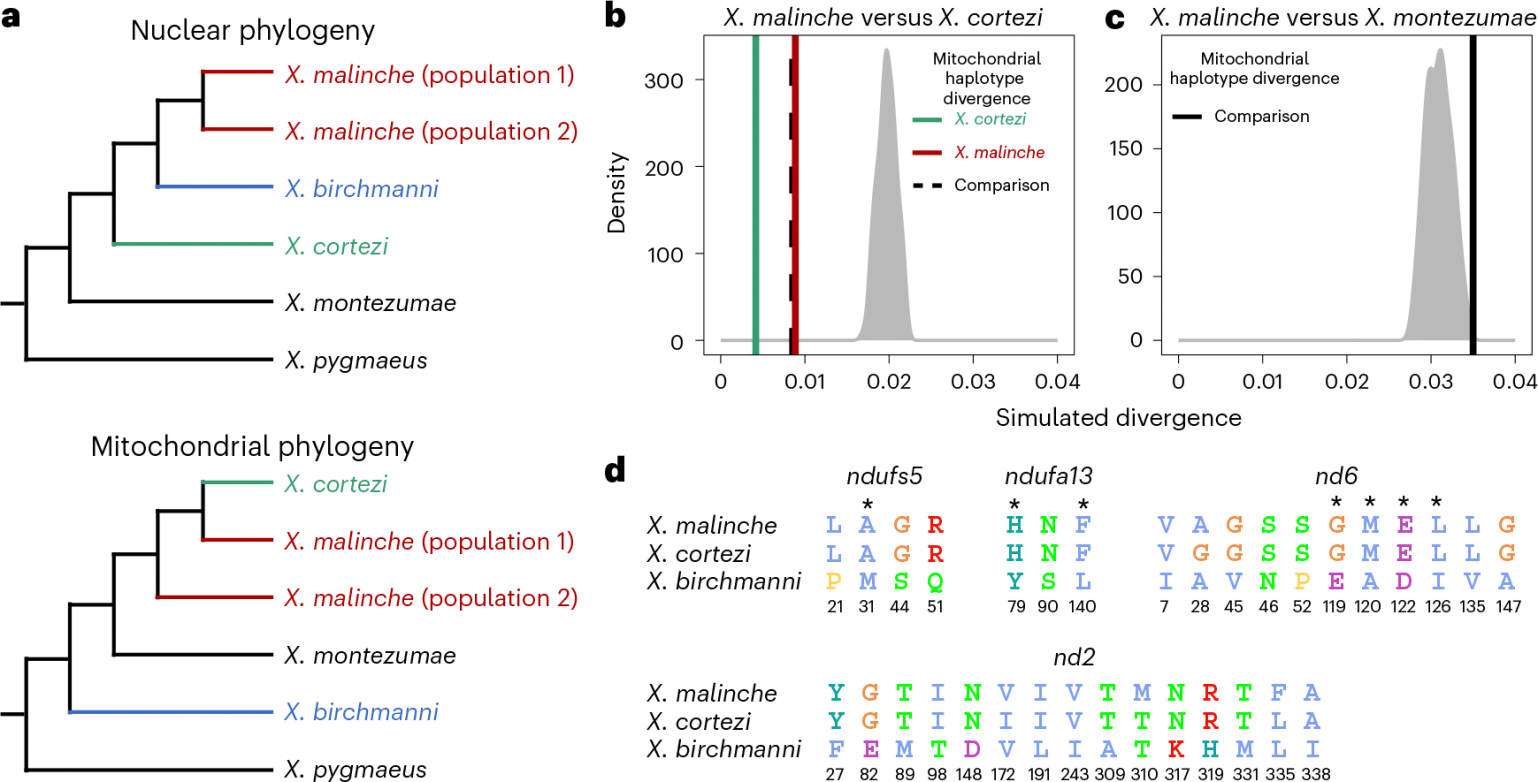
Genetic relationships and mitochondrial divergence between *X. birchmanni*, *X. cortezi* and *X. malinche.* **a**, Nuclear^35,51^ and mitochondrial phylogenies show discordant topologies that reflect ancient hybridization between *X. malinche* (red) and *X. cortezi* (green), resulting from introgression of the mitochondria from *X. malinche* into *X. cortezi*. See Extended Data Fig. 7 for an expanded mitochondrial phylogeny. **b**, Simulations confirm that *X. malinche* and *X. cortezi* have much lower mitochondrial sequence divergence than expected in a scenario lacking gene flow. The density plot shows expected mitochondrial haplotype divergence across 100 replicate simulations modelling divergence between *X. malinche* and *X. cortezi*. The green line shows average pairwise mitochondrial haplotype divergence between different *X. cortezi* populations, the red line shows average pairwise mitochondrial haplotype divergence between different *X. malinche* populations and the dashed line shows average pairwise mitochondrial haplotype divergence between *X. cortezi* and *X. malinche*. **c**, By contrast, *X. malinche* does not have lower than expected mitochondrial sequence divergence in comparisons to another closely related species, *X. montezumae*. The density plot shows expected mitochondrial haplotype divergence across 100 replicate simulations modelling divergence between *X. malinche* and *X. montezumae*. The black line shows observed mitochondrial haplotype divergence between *X. malinche* and *X. montezumae*. **d**, Amino acid differences between *X. malinche*, *X. cortezi* and *X. birchmanni* at *ndufs5*, *ndufa13* and mitochondrially encoded proteins *nd6* and *nd2*. Protein modelling results indicate that these proteins are in close physical contact in mitochondrial protein complex I, with several instances of physical contact between substitutions in *X. birchmanni* and *X. malinche/X. cortezi* at the interface of *ndufs5*, *ndufa13* and *nd6* (Supplementary Fig. 9 and ref. 35). Asterisks indicate substitutions at predicted points of protein–protein contact between *ndufs5*, *ndufa13* and *nd6*, and colours follow the Clustal2 amino acid colour scheme.

Recent work in our group identified two genes involved in a lethal genetic incompatibility between the nuclear genome of *X. birchmanni* at *ndufs5* (NADH dehydrogenase ubiquinone iron-sulfur protein 5) and *ndufa13* (NADH dehydrogenase ubiquinone 1 alpha subcomplex subunit 13) and the mitochondrial genome of its sister species, *Xiphophorus malinche*^35^. The genes *ndufs5* and *ndufa13* physically colocalize in mitochondrial protein complex I and contact multiple mitochondrially encoded proteins^35^. Interestingly, the previous results suggested the possibility that all components of this incompatibility are also present in *X. cortezi* due to historical introgression of the *X. malinche* mitochondria, *ndufs5* and *ndufa13* (Fig. 3a). We confirmed this pattern with a phylogenetic analysis using a large and geographically diverse sampling (Supplementary Discussion 8). We found clear evidence that *X. cortezi* mitochondrial diversity is clustered within*X. malinche* diversity (Extended Data Fig. 7), differing from the nuclear relationships^51^ (Fig. 3a). Moreover, simulations confirmed that sequence divergence between *X. malinche* and *X. cortezi* mitochondrial haplotypes was markedly lower than expected in a scenario of divergence without gene flow (Fig. 3b and Supplementary Discussion 8)^35^. Notably, mitochondrial divergence between *X. malinche* and *X. cortezi* is similar to observed divergence across different *X. malinche* populations (Fig. 3b). By contrast, both species have roughly expected levels of mitochondrial sequence divergence to another closely related species, *Xiphophorus montezumae* (Fig. 3c). However, both *X. malinche* and *X. cortezi* have much greater than expected mitochondrial divergence from *X. birchmanni* (Supplementary Fig. 7), potentially pointing to additional complexity in mitochondrial evolution in this species (Supplementary Discussion 8). Additional simulations estimating the divergence time between *X. malinche* and *X. cortezi* mitochondrial haplotypes find evidence of a more recent estimated divergence time compared to the nuclear divergence of ~450,000 generations^32,42^ (Supplementary Fig. 8; maximum a posteriori (MAP) estimate = 202,320 generations, 95% credible interval (CI) = 165,288–231,918).

Using geographically diverse samples, we determined that *X. malinche* and *X. cortezi* have identical amino acid sequences at *ndufs5* and *ndufa13*, and differ from *X. birchmanni* at the same non-synonymous substitutions: four in *ndufs5* and three in *ndufa13* (Fig. 3d, see also ref. 35). Moreover, *X. malinche* and *X. cortezi* have nearly identical amino acid sequences at the mitochondrially encoded proteins which interact with *ndufs5* and *ndufa13*, *nd6* (NADH-ubiquinone oxidoreductase chain 6) and *nd2* (NADH-ubiquinone oxidoreductase chain 2), and both differ dramatically from *X. birchmanni* (Fig. 3d and Supplementary Fig. 9a). The only substitutions present between *X. malinche* and *X. cortezi* in the *nd6* and *nd2* proteins fall outside their interface with *ndufs5* and *ndufa13* (Supplementary Fig. 9b).

**Fig. 4 |:**
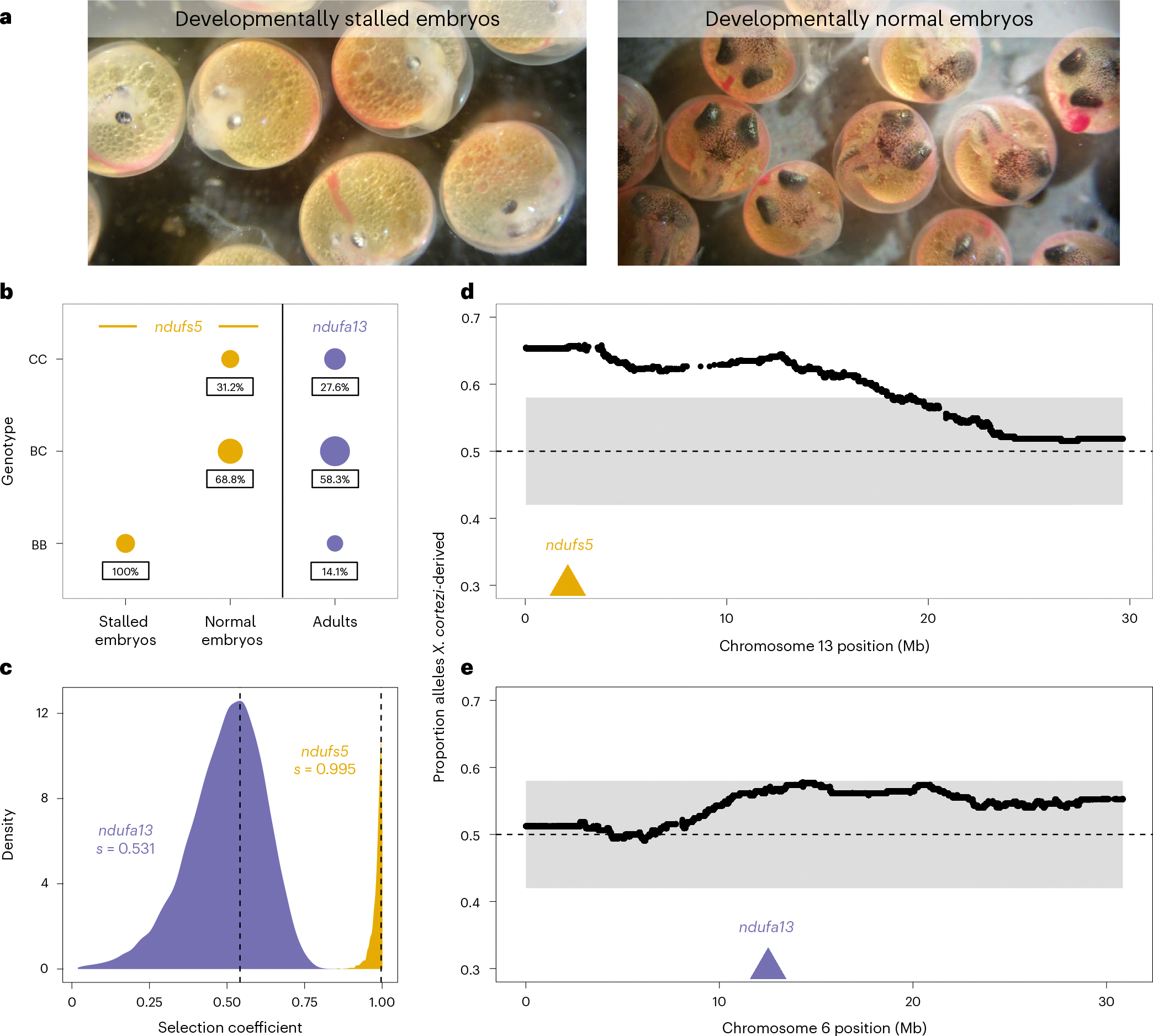
Characterization of a genetic incompatibility involving the *X. cortezi* mitochondrial genome identified using laboratory-generated F_2_ hybrids. **a**, F_2_ embryos dissected from pregnant females exhibit two phenotypes: development that stalls at an early stage (left) or normal development (right). All pictured embryos are siblings from the same brood taken on the same day. All F_2_ embryos possess the mitochondrial haplotype introgressed from *X. malinche*. **b**, All embryos that have developmentally stalled possess the homozygous *X. birchmanni* genotype at *ndufs5* (two-sided Chi-squared test: *n* = 33, *χ*^2^ = 99, *P* = 3.18 × 10^−22^), while the normally developing embryos only possess the other two genotypes (*n* = 93, *χ*^2^ = 31.258, *P* = 1.63 × 10^−7^). Moreover, few F_2_ adults possess the homozygous *X. birchmanni* genotypes for *ndufa13*, strongly differing from expected genotype frequencies under Mendelian inheritance (*n* = 163, *χ*^2^ = 10.411, *P* = 0.0055). See Extended Data Fig. 8 for *ndufa13* results in embryos. Point sizes indicate the number of samples and values underneath each point indicate the percentage of samples within a development group that possessed a particular genotype. Genotypes: CC, homozygous *X. cortezi*; BC, heterozygous; BB, homozygous *X. birchmanni*. Expected genotype frequencies in this cross are 25% CC, 50% BC and 25% BB. **c**, Results of ABC simulations to estimate the strength of selection on *ndufs5* (yellow) and *ndufa13* (purple). Density plots show the posterior distribution from accepted ABC simulations and the dashed line and text indicate the MAP estimate of the selection coefficient (*s*). Incompatible interactions at both genes are inferred to be largely recessive (*ndufs5* MAP estimate *h* = 0.027, Extended Data Fig. 9a; *ndufa13* MAP estimate *h* = 0.049, Extended Data Fig. 9b). **d**,**e**, Average ancestry of F_2_ adults reveals segregation distortion that surpasses our 95% simulated genome-wide significance threshold (grey envelope) on chromosome 13 near *ndufs5* and approaches our significance threshold (**d**) on chromosome 6 near *ndufa13* (**e**). The locations of *ndufs5* and *ndufa13* are indicated with triangles. The dashed line at 0.5 represents the expected *X. cortezi* ancestry in this cross. See Supplementary Fig. 11 for a representative chromosome that lacks segregation distortion in F_2_ hybrids.

Together, these data indicate that we should expect *X. cortezi* × *X. birchmanni* hybrids to suffer from the same mitonuclear incompatibility identified in *X. malinche* × *X. birchmanni* hybrids. To directly test for the presence of this genetic incompatibility, we used F_1_s from the successful cross direction to produce F_2_s (the cross design means all F_2_s possess the *X. cortezi* mitochondria). We first characterized developing F_2_ embryos from four pregnant F_1_ females (*n* = 127) and consistently found two types of F_2_ embryos (Fig. 4a): 26.0% had stalled at an early stage of development (around stage 7 out of 11; ref. 52), while the remaining continued to develop beyond this stage. Note that fertilization within a brood is nearly simultaneous in *Xiphophorus* and that appreciable developmental lag is never observed in pure species^35^. The stalled embryos had a smaller body size with smaller head/eyes relative to body length and appeared to have reduced vasculature in the yolk in comparison to the remaining embryos (Fig. 4a and Supplementary Fig. 10). Using whole-genome sequencing and local ancestry inference, we genotyped embryos and determined whether they were homozygous *X. birchmanni*, homozygous *X. cortezi* or heterozygous at ancestry informative sites within *ndufs5* and *ndufa13*. We found striking patterns for *ndufs5* (Fig. 4b), with all developmentally stalled embryos possessing the homozygous *X. birchmanni* genotype (two-sided Chi-squared test: *χ*^2^ = 99, *P* = 3.18 × 10^−22^), while all remaining embryos were either heterozygous or homozygous *X. cortezi* (*χ*^2^ = 31.258, *P* = 1.63 × 10^−7^). Remarkably, this is exactly the phenotype observed in *X. malinche* × *X. birchmanni* hybrids that possess the *ndufs5* incompatibility^35^.

**Extended Data Fig. 8 |:**
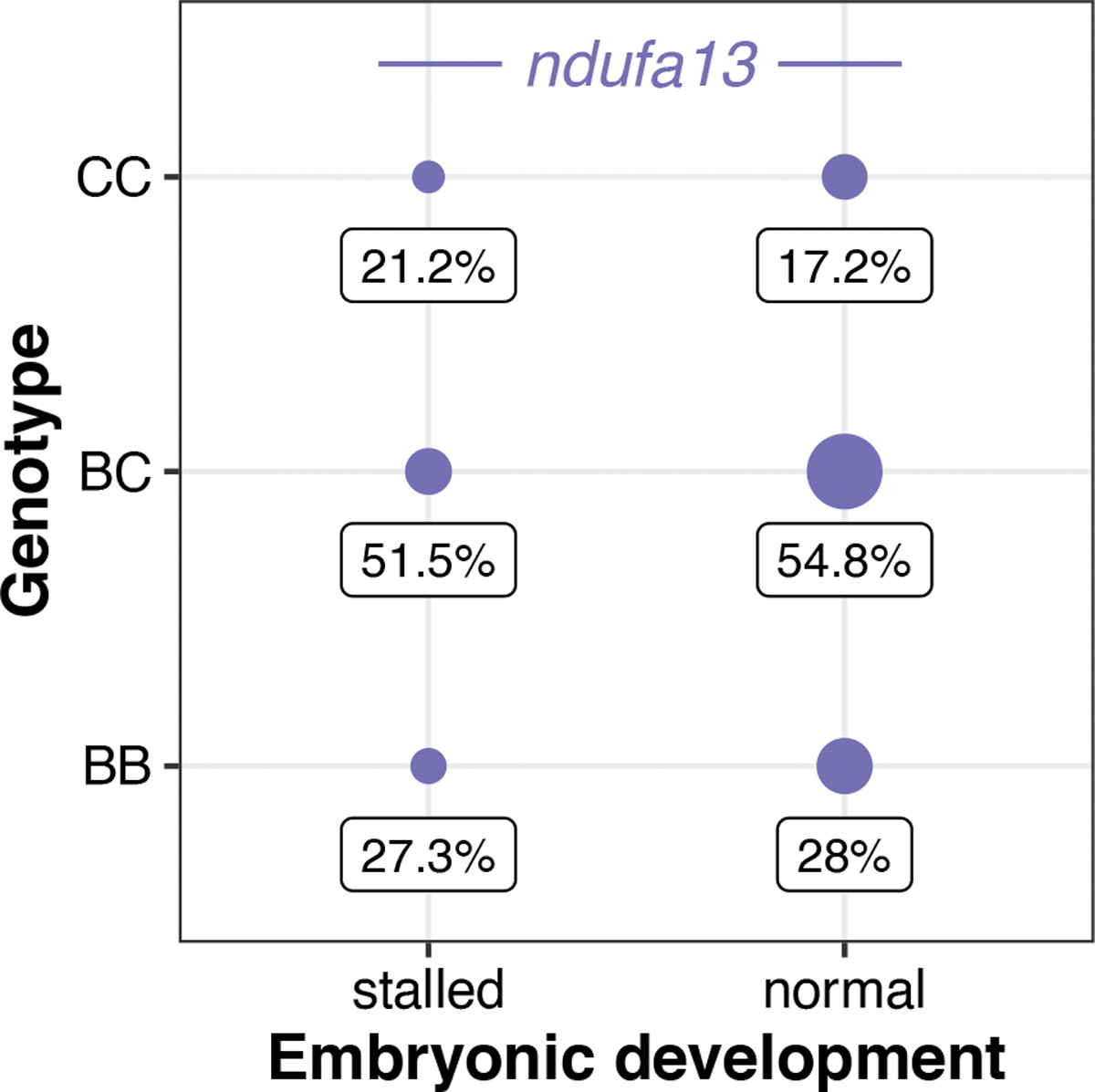
*ndufa13* genotypes present in F_2_ embryos. All genotypes at *ndufa13* were present in both groups of F_2_ embryos and did not differ from Mendelian expectations for the developmentally stalled (two-sided Chi-squared test: *n* = 33, χ^2^ = 3.021, *P* = 0.2207) or developmentally normal (*n* = 93, χ^2^ = 0.273, *P* = 0.8725) embryos. Point sizes indicate the number of samples and values underneath each point indicate the percent of samples for each developmental stage that possessed a particular genotype. Genotypes: CC = homozygous *X. cortezi*, BC = heterozygous, BB = homozygous *X. birchmanni*. Expected genotype frequencies in this cross are 25% CC, 50% BC, and 25% BB.

Ancestry mismatch at *ndufa13* in *X. malinche* × *X. birchmanni* hybrids does not impact embryonic development but instead causes lethality postbirth^35^. Consistent with this, *X. birchmanni* ancestry at *ndufa13* in *X. cortezi* × *X. birchmanni* F_2_ embryos did not deviate from expectations, regardless of whether the embryos were developmentally stalled (*χ*^2^ = 3.021, *P* = 0.2207) or not (*χ*^2^ = 0.273, *P* = 0.8725; Extended Data Fig. 8). However, we found disproportionate early-life lethality as we tracked F_2_s through postembryonic development, such that we found a lack of adult F_2_s that possessed the homozygous *X. birchmanni* genotype of *ndufa13* (Fig. 4b).

**Extended Data Fig. 9 |:**
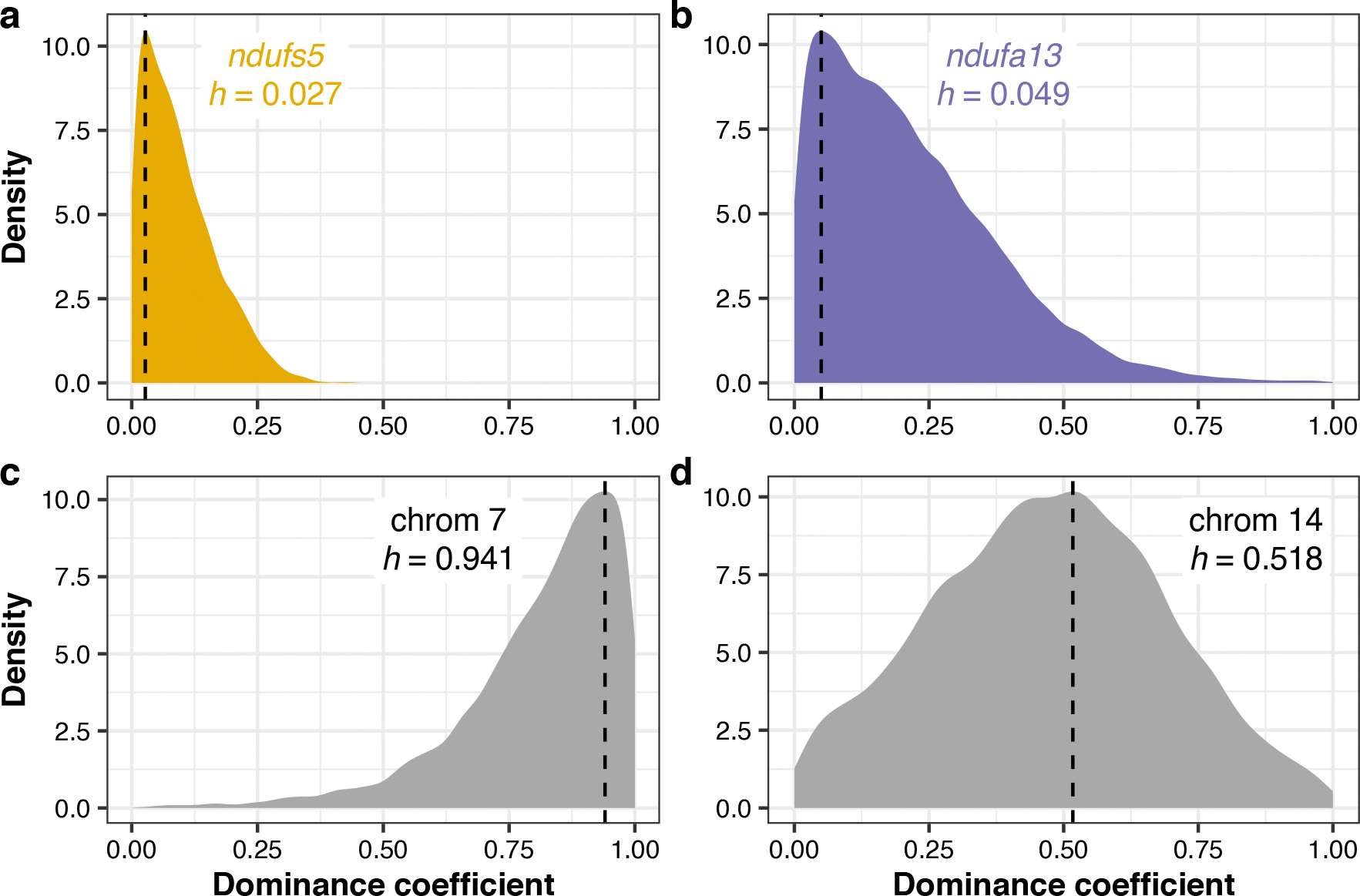
Additional parameters from ABC simulations of regions under strong selection in F_2_s. Posterior distributions of dominance coefficients (*h*) from approximate Bayesian computation (ABC) simulations of (**a**) *ndufs5*, (**b**) *ndufa13*, and regions under selection on (**c**) chromosome 7 and (**d**) chromosome 14. Density plots show the posterior distribution from accepted ABC simulations and the dashed line and text indicate the maximum a posteriori estimate for the dominance coefficient. Corresponding results for the selection coefficient are shown in Fig. 4c and Fig. 5.

Among F_2_s that survived to adulthood (*n* = 163), we found segregation distortion beyond our simulated 95% significance threshold (Supplementary Fig. 11 and Supplementary Discussion 9) on chromosome 13 near *ndufs5* (Fig. 4d) and approaching significance on chromosome 6 near *ndufa13* (Fig. 4e). We found a striking lack of adult F_2_s with homozygous *X. birchmanni* genotypes at these genes, such that both *ndufs5* (*χ*^2^ = 55.025, *P* = 1.13 × 10^−12^) and *ndufa13* (*χ*^2^ = 10.411, *P* = 0.0055; Fig. 4b) strongly differ from expected genotype frequencies in adults. Using approximate Bayesian computation (ABC) simulations and observed ancestry data from surviving F_2_s, we inferred the strength of selection against *X. birchmanni* ancestry consistent with observed patterns at *ndufs5* and *ndufa13*. We found selection against *X. birchmanni* ancestry at *ndufs5* in F_2_s to be largely recessive and nearly complete (Fig. 4c, MAP estimate of selection coefficient *s* = 0.995, 95% CI *s* = 0.933–1.000; Extended Data Fig. 9a, MAP estimate of dominance coefficient *h* = 0.027, 95% CI *h* = 0.004–0.267). Notably, this estimated *s* mirrors that inferred for *X. malinche* × *X. birchmanni* hybrids for the same genetic interaction (MAP estimate *s* = 0.996, 95% CI *s* = 0.986–0.999)^35^. Although weaker than selection on *ndufs5*, the strength of selection against *X. birchmanni* ancestry at *ndufa13* is also quite strong (Fig. 4c, MAP estimate *s* = 0.531, 95% CI *s* = 0.201–0.694; Extended Data Fig. 9b, MAP estimate *h* = 0.049, 95% CI *h* = 0.008–0.606). Intriguingly, selection is substantially weaker than inferred for the same genetic interaction in *X. malinche* × *X. birchmanni* hybrids, even accounting for differences in power (Supplementary Discussion 10).

### Additional evidence for postzygotic selection

**Extended Data Fig 10 |:**
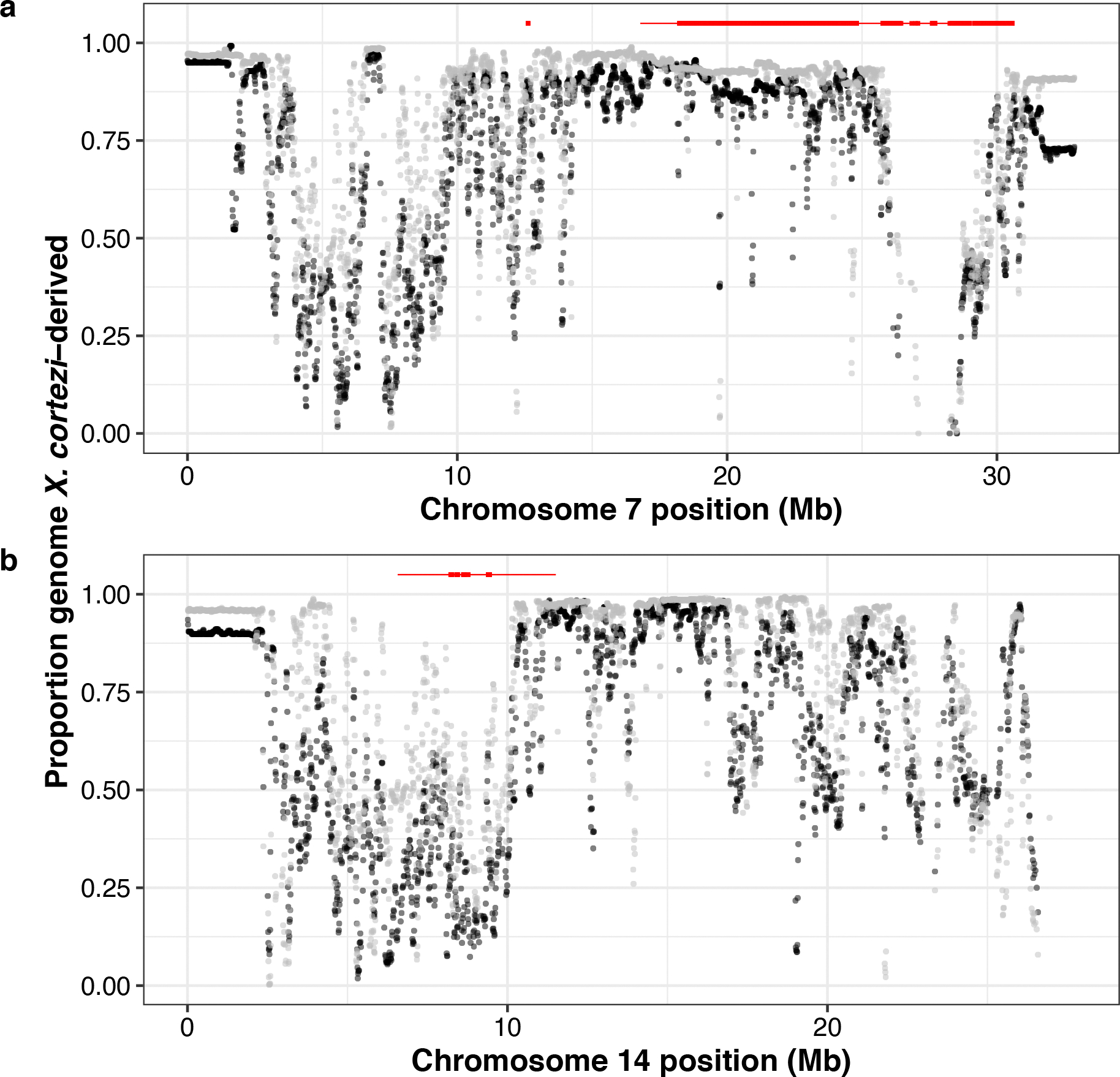
Ancestry of newly identified genomic regions in two hybrid populations. Comparison of *X. cortezi* ancestry in 10 kb windows on (**a**) chromosome 7 and (**b**) chromosome 14 in admixed cluster individuals from the Río Santa Cruz (gray) and Chapulhuacanito (black) hybrid populations using data collected in a companion study^43^. The regions that cross the genome-wide significance threshold of expected ancestry given the cross design (Fig. 5), along with the regions they are in strong LD with, are highlighted in red at the top of the plot (significant regions as a thick line and regions in strong LD with a thin line). Both regions on chromosome 7 and 14 span regions of elevated *X. cortezi* ancestry, as well as regions with elevated *X. birchmanni* ancestry.

**Fig. 5 |:**
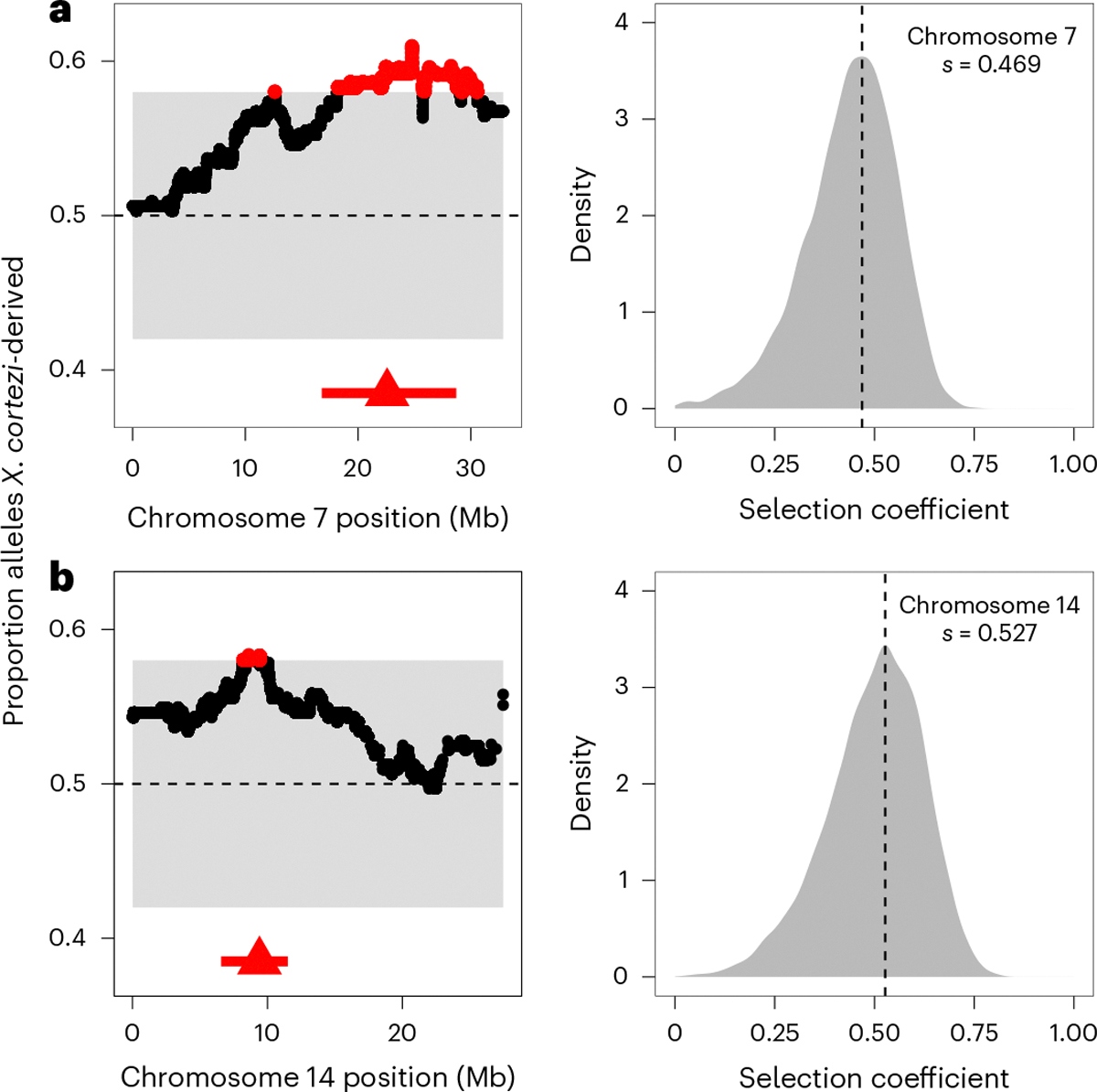
Identification of two additional genomic regions under selection in *X. cortezi* × *X. birchmanni* hybrids. **a**,**b**, Left: regions on chromosome 7 (**a**) and chromosome 14 (**b**) have average ancestry of F_2_ adults that surpass (red points) our 95% simulated significance threshold (grey envelope). The dashed line at 0.5 represents the expected *X. cortezi* ancestry from this cross. The genomic positions used for ABC simulations are shown with red triangles and the regions in strong LD with these positions in our F_2_ population are indicated by a red line. See Supplementary Fig. 11 for a representative chromosome that lacks segregation distortion. Right: results of ABC simulations to infer the strength of selection on the indicated regions on chromosome 7 (**a**) and chromosome 14 (**b**). Density plots show the posterior distribution from accepted ABC simulations and the dashed line and text indicate the MAP estimate for the selection coefficient (*s*). Both the regions on chromosome 7 (**a**) and chromosome 14 (**b**) are inferred to be partially dominant (chromosome 7—MAP estimate *h* = 0.941, Extended Data Fig. 9c; chromosome 14—MAP estimate *h* = 0.518, Extended Data Fig. 9d).

We evaluated evidence for other selection genome-wide in hybrids using local ancestry results from our F_2_ cross. With 163 F_2_s, we expected to have only moderate power to detect loci under strong selection (Supplementary Fig. 12 and Supplementary Discussion 9). However, in addition to the incompatibility described above, we identified two more regions on chromosomes 7 and 14 that significantly deviated from the expected 50–50 ancestry proportion (Fig. 5 and Supplementary Tables 5 and 6). Since all F_2_s were laboratory-raised, this suggests the presence of additional incompatibilities between the *X. cortezi* and *X. birchmanni* genomes that impact viability or F_1_ fertility in the laboratory environment. We estimate the strength of selection against *X. birchmanni* ancestry to be 0.469 (95% CI *s* = 0.177–0.626) and 0.527 (95% CI *s* = 0.229–0.703) on the regions on chromosomes 7 and 14, respectively (Fig. 5). Fitness effects of *X. birchmanni* ancestry are inferred to be partially dominant in both cases (Extended Data Fig. 9c,d; chromosome 7—MAP estimate *h* = 0.941, 95% CI *h* = 0.398–0.994; chromosome 14—MAP estimate *h* = 0.518, 95% CI *h* = 0.055–0.892). Notably, each of these genomic regions spans areas of both strongly elevated and strongly reduced *X. cortezi* ancestry in two independent hybrid populations (Extended Data Fig. 10)^43^. Although we have not yet identified the drivers of selection, some of the genes within both regions directly interact with the mitochondria (Supplementary Tables 5 and 6) and will serve as interesting candidates for future work (Supplementary Discussion 11).

## Discussion

Decades of study have investigated how diverse isolating mechanisms can limit gene flow between species^1^. However, despite evidence that such isolating mechanisms are common, even in recently diverged lineages^53,54^, genomic sequencing has provided extensive evidence that hybridization is also widespread^3,4^. How do we reconcile the frequency of hybridization over evolutionary time with the evidence that reproductive barriers between diverging lineages are ubiquitous? Here, we show multiple barriers to gene flow impacting mating and viability in closely related swordtails, yet also find that much of the genome is porous to genetic exchange in natural hybrid populations. Moreover, we provide one of the most comprehensive descriptions of the way in which hybridization itself could lead to reproductive isolation: we demonstrate that genes that cause a genetic incompatibility actively reduce gene flow in multiple species when spread through ancient hybridization. This finding adds a new dimension to our understanding of the interplay between hybridization and the evolution of reproductive barriers.

Combining whole-genome sequencing with developmental and behavioural assays, we investigated reproductive barriers between *X. birchmanni* and *X. cortezi* to disentangle the role of prezygotic and postzygotic mechanisms in limiting gene flow. We identified a strongly bimodal distribution in ancestry in a newly identified hybrid population, Chapulhuacanito (Fig. 1). This is strikingly similar to the pattern we previously observed in an independently formed hybrid population in the Río Santa Cruz^42^, highlighting surprising repeatability in hybrid population evolution in this system. Interestingly, this bimodality has been present since at least 2003 in Chapulhuacanito (Fig. 1c). The consistency over time and the similarity across replicated hybrid populations suggests that the outcomes of hybridization between these species at the genome-wide scale are, in part, predictable.

What mechanisms drive this bimodal population structure? Assortative mating, a prezygotic mechanism, may influence reproductive isolation in *X. birchmanni* and *X. cortezi* and maintain the observed population structure. Notably, assortative mating has previously been implicated in the bimodal ancestry distribution of a hybrid population between *X. birchmanni* and its sister species *X. malinche*^46^. By assessing genome-wide ancestry of wild-caught females and their embryos, we found that prezygotic reproductive barriers are indeed strong in Chapulhuacanito (Fig. 2a). We found no evidence of fertilization by males from the alternative ancestry cluster and simulations suggested that assortative mating with same-ancestry individuals approached 100%. Work in the independent hybrid population between these two species in the Río Santa Cruz has shown similarly strong assortative mating^42^. However, our population-level sampling highlights that cross-cluster matings do occur at low frequencies (Extended Data Fig. 1), indicating that assortative mating does not completely prevent gene flow (consistent with findings in other systems^21,31,55^).

Using in-laboratory behavioural assays, we tested the presence and strength of female preferences in explaining these assortative mating patterns. Across our trials, we found a complex suite of results (Fig. 2b and Extended Data Fig. 3). While *X. cortezi* females showed preferences for conspecific males when exposed only to visual cues, this preference was lost if females could also assess pheromone cues. The *X. birchmanni* females did not show behavioural evidence of assortative mating in any of our assays. However, in separate experiments we did find strong differences in behaviour that indicate a relationship between genome-wide *X. cortezi* ancestry and increased boldness (Extended Data Fig. 4). It is possible that this increased boldness in the laboratory translates into different habitat use in the wild, although results from our collections and underwater recordings suggest substantial overlap in habitat use at Chapulhuacanito (Supplementary Discussion 2). The results of our behavioural assays underscore complex interactions between behaviour, assortative mating dynamics and other reproductive barriers (Supplementary Discussion 5). Moreover, we note that major differences in sperm morphology and motility between species and hybrids (Extended Data Figs. 5 and 6) may also contribute to reproductive barriers through cryptic female choice.

While we detected conspecific behavioural preferences in *X. cortezi* females, we found no evidence for such preferences in *X. birchmanni* females. We were initially surprised by this result since individuals in the *X. birchmanni* cluster had near-zero levels of introgression from *X. cortezi* in both independently formed hybrid populations between the two species, Chapulhuacanito (Fig. 1) and the Río Santa Cruz^42^. This suggested the potential presence of a strong postmating barrier when this cross involves *X. birchmanni* females and, indeed, we found nearly complete developmental inviability in the laboratory F_1_ cross with *X. birchmanni* mothers (Supplementary Discussion 6). Importantly, this pattern of developmental inviability provides a natural explanation for the repeatable absence of introgression into the *X. birchmanni* cluster across natural hybrid populations.

Crosses between *X. cortezi* mothers and *X. birchmanni* fathers frequently resulted in viable and fertile offspring, consistent with higher levels of admixed ancestry in the admixed cluster hybrids in natural populations. However, we found that this cross direction also showed signs of strong postzygotic reproductive barriers. F_1_ offspring had a strikingly skewed sex ratio with about six females for every one male produced. Moreover, we detected strong evidence for segregation distortion consistent with hybrid inviability across the genomes of F_2_s (see below; Figs. 4 and 5) and unusual sperm morphology in F_1_s and F_2_s compared to the parental species (Extended Data Fig. 5). Surprisingly, even in the presence of assortative mating and these diverse postzygotic barriers, much of the genome of *X. cortezi* appears to be permeable to introgression from *X. birchmanni*. This result highlights how the presence of diverse reproductive barriers is not irreconcilable with the general finding that many species have derived substantial proportions of their genome from hybridization.

Our results also add new complexity to the understanding of how historical gene flow itself interfaces with present-day reproductive isolation. Ancient hybridization between *X. cortezi* and *X. malinche* (Fig. 3) has led to introgression of the *X. malinche* mitochondria and two interacting genes, *ndufs5* and *ndufa13*, into the *X. cortezi* lineage. Together with mitochondrially encoded proteins, *ndufs5* and *ndufa13* form a large protein complex in the essential mitochondrial electron transport chain. These genes are involved in a lethal mitonuclear incompatibility between *X. malinche* and *X. birchmanni*^35^, driven by combining the *X. malinche* mitochondria with the *X. birchmanni* alleles of *ndufs5* and *ndufa13*. We show here that the same loci cause incompatibility between *X. cortezi* × *X. birchmanni* hybrids and that the phenotypic consequences of incompatible genotypes (Fig. 4a) are strikingly similar to those observed in *X. malinche* × *X. birchmanni* hybrids^35^. Previous work in a diversity of systems^24–27^ has hinted at the role that complex historical gene flow might play in influencing reproductive isolation. Here, we provide an empirical demonstration that genes involved in an incompatibility can result in remarkably similar consequences for reproductive isolation when introgressed into a third species. These incompatibilities also shape ancestry patterns in natural hybrid populations. In a companion study, we found that admixed cluster individuals in the two independent hybrid populations have genomic ‘deserts’ where *X. birchmanni* ancestry is extraordinarily depleted from these regions of the genome^43^. While the full consequences of this ancient introgression event are still unclear, our results illustrate how past gene flow can impact present-day patterns of reproductive isolation, especially in species groups where hybridization occurs between multiple lineages (Supplementary Discussion 12).

In this study, we find that multiple, overlapping prezygotic and postzygotic barriers to gene flow result in strong but incomplete reproductive isolation between *X. cortezi* and *X. birchmanni*. We describe how assortative mating, hybrid inviability, genetic incompatibilities and ancient introgression all contribute to the overall level of reproductive isolation between these swordtails and document how this leads to repeatability in evolution at the population level in hybrid populations. Additionally, our results support the surprising finding that ancient introgression moved genes that are now involved in strong genetic incompatibilities across species boundaries. Our results open a compelling new avenue of both empirical and theoretical research exploring previously unappreciated roles that hybridization may play in the evolution of reproductive isolation.

## Methods

### Sample collection

Natural hybrids were collected from the Chapulhuacanito population (21° 12′ 10.58″ N and 98° 40′ 28.27″ W) using baited minnow traps (*n* = 306). Each fish was anaesthetized in 100 mg ml^−1^ MS-222 and river water before being photographed. A small fin clip was taken from the upper caudal fin of each individual and preserved in 95% ethanol for DNA extraction. Fish were allowed to recover in river water before being released at the collection site. A subset of pregnant females from Chapulhuacanito (*n* = 49) were euthanized in an overdose of MS-222 and preserved in 95% ethanol for paired mother/embryo sequencing (see details below). Collections were conducted with permission from the Mexican government (permit no. PPF/DGOPA-002/19).

We also took advantage of historical samples from 2003 (*n* = 11), 2006 (*n* = 21) and 2017 (*n* = 41) at Chapulhuacanito collected through a companion study^43^. These samples were preserved in either dimethyl-sulfoxide or 95% ethanol at the time of collection.

### DNA extraction and low-coverage library preparation

DNA was extracted from fin clips and embryos using the Agencourt DNAdvance magnetic bead-based purification system (Beckman Coulter) in a 96-well plate format. We followed the recommended protocol for extraction from tissue except that we used half-reactions. Following extraction, DNA was quantified with a BioTek Synergy H1 microplate reader (Agilent Technologies) and diluted to a concentration of 2.5 ng μl^−1^. We prepared libraries for low-coverage whole-genome sequencing using a tagmentation-based protocol and the Illumina Tagment DNA TDE1 Enzyme and Buffer Kit (Illumina). Briefly, samples were enzymatically sheared and initial adaptors were added by incubation with the tagmentation enzyme and buffer at 55 °C for 5 min. Individual i5 and i7 indices were added via a polymerase chain reaction (PCR) using the OneTaq HS Quick-Load 2X Master Mix (New England Biolabs). Following PCR, samples were pooled and purified using 18% SPRI magnetic beads, quantified with a Qubit Fluorometer (Thermofisher Scientific) and visualized on a Tapestation 4200 (Agilent Technologies). Pooled libraries were sequenced on either an Illumina HiSeq 4000 or Illumina NovaSeq X Plus at Admera Health Services.

### Global and local ancestry inference

We used a newly developed local ancestry inference^41^ pipeline to infer ancestry across the genome of sampled individuals^32,42,43^. While we describe this pipeline in extensive detail in our companion study^43^, we explain the approach briefly here. The most recent version of this pipeline uses chromosome-scale assemblies for *X. birchmanni* and *X. cortezi* generated with PacBio HiFi data. Using sequencing of several allopatric *X. birchmanni* and *X. cortezi* populations and artificially produced F_1_ hybrids, we identified 1,001,684 ancestry informative sites that are fixed or nearly fixed between species^32,42,43^. For all sequenced individuals, we map low-coverage (~1X) whole-genome sequencing data to both the *X. birchmanni* and *X. cortezi* reference genomes and generate a table of counts at ancestry informative sites. While low-coverage data will often fail to capture both alleles at a given site heterozygous for the two ancestry states, because of admixture linkage disequilibrium in hybrids, ancestry states are correlated over tens of thousands to hundreds of thousands of basepairs. Thus, by applying a hidden Markov model to these counts, we can accurately infer ancestry along the genome. Past work using both simulations^41^ and the results of artificial F_2_ crosses^43^ has shown that this approach is extremely accurate for inferring local ancestry in *X. cortezi* × *X. birchmanni* hybrids (for example, Supplementary Figs. 13 and 14), with estimated error rates of <0.1% per ancestry informative site.

We ran the ancestryinfer pipeline on individuals from Chapulhuacanito using priors for the time since initial admixture set to 50 and the genome-wide admixture proportion set to a uniform prior of 0.5 (Supplementary Discussion 13 and Supplementary Fig. 15 discuss the impact of using a uniform admixture prior). The output of this pipeline is posterior probabilities of each ancestry state at every ancestry informative site that distinguishes *X. birchmanni* and *X. cortezi* throughout the genome. At a given ancestry informative site, if an individual had >0.9 posterior probability for a given ancestry state (for example, homozygous *X. birchmanni*, heterozygous or homozygous *X. cortezi*), we converted the ancestry at the site to that ancestry state. For sites where no ancestry state had >0.9 posterior probability, we converted the site to NA (not applicable).

For a given individual, this allowed us to estimate the proportion of the genome derived from each parental species, as well as determine ancestry at individual sites of interest along the genome. To examine ancestry at genes that had previously been implicated in mitonuclear incompatibilities^35^, we selected an ancestry informative site that fell within the gene of interest and was covered in the most individuals. In cases where multiple sites satisfied these criteria, we randomly selected a site. Because of high admixture linkage disequilibrium (LD) in hybrids, the correlation in ancestry across sites within a gene is extremely high. Because the number of ancestry informative sites differs between genes, we considered it less biased to randomly subsample a single ancestry informative site for each.

### Artificial crosses

To produce F_1_s, we seeded 2,000-l outdoor mesocosms with wild-caught adults from allopatric populations: *X. cortezi* from Puente de Huichihuayán (21° 26′ 9.95″ N and 98° 56′ 0.00″ W) and *X. birchmanni* from Coacuilco (21° 5′ 51.16″ N and 98° 35′ 20.10″ W). We expected we might find differences in cross success depending on the sex of each species used^56^, so we set up crosses in both directions with a 1:3 male to female sex ratio. Because *Xiphophorus* can store sperm and the adults were wild-caught, all offspring were collected and sequenced to identify resulting F_1_s. Male and female F_1_s were subsequently crossed by randomly seeding a 567-l outdoor mesocosm with a 1:3 male to female sex ratio to produce F_2_s.

F_2_ offspring (*n* = 163) were collected soon after birth and raised in small groups in indoor tanks. Once they were large enough (~2–3 months old), individuals were marked with elastomer tags and fin clipped. We extracted DNA from these fin clips, performed library preparation and local ancestry inference as described above, except that we set the prior for the time since initial admixture to 2. Regions of significant segregation distortion were defined as those that exceeded our expectations for average ancestry based on simulations of F_2_ hybrids (Supplementary Discussion 9).

Because our sample size is relatively small and our power to detect selection is modest (Supplementary Fig. 12 and Supplementary Discussion 9), we chose to define the interval of interest for segregation distortion analyses based on LD, rather than simply focusing on markers that surpass the segregation distortion threshold. This addresses the possibility that variance in missing data could impact the intervals we define as segregation distorters. LD decay in the parental species occurs over ~50 kilobases (kb); however, in laboratory-produced hybrids admixture LD stretches for many megabases. We thinned our ancestry calls to retain one ancestry informative site per 50 kb, and then converted our calls to PLINK format. Thinning to retain one marker is for computational tractability, but since F_2_ individuals harbour approximately two crossovers per chromosome (or two per ~2,500 kb), this does not result in any information loss relative to the full dataset. Next, we used PLINK to calculate *R*^2^ between the peak segregation distortion marker and other sites on the same chromosome. We then determined the distance over which *R*^2^ fell below 0.8 in either direction of the peak marker and treated this as our segregation distortion interval of interest.

### Approximate Bayesian computation to infer selection strength

Once we had identified regions with significant segregation distortion in F_2_ hybrids, we wanted to infer the strength of selection on these regions consistent with patterns observed in the empirical data. To do so, we used population genetic models of Hardy–Weinberg equilibrium with selection. For *ndufa13* (chromosome 6) and *ndufs5* (chromosome 13), the known partner genes in the hybrid incompatibility are mitochondrially encoded. Since all F_2_ hybrids had *X. cortezi* mitochondria, we simply modelled selection against the *X. birchmanni* alleles at *ndufa13* and *ndufs5*. For each simulation, we drew a selection coefficient and dominance coefficient from a random uniform distribution ranging from 0 to 1. We modified the expected genotype frequencies in adult F_2_s from those expected at fertilization based on the simulated values of *s*, the selection coefficient, and the dominance coefficient (*h*). We then used these expected frequencies after selection to draw genotypes for 163 individuals (equal to our F_2_ sample size). As summary statistics, we used the average *X. birchmanni* ancestry at the selected site and the number of individuals heterozygous or homozygous for *X. birchmanni* ancestry. We accepted simulations that fell within 5% of the observed data and used these accepted simulations to generate posterior distributions of *s* and *h*.

For loci on chromosomes 7 and 14, we do not know the mechanisms of selection acting on them (that is, whether they represent loci involved in nuclear–nuclear or nuclear–mitochondrial incompatibilities or some other mechanism of selection on hybrids; Supplementary Discussion 11). Inspection of genotypes in both regions indicates that they are depleted in homozygous *X. birchmanni* ancestry, so we chose to estimate *s* and *h* in the same way as described above. We note that if *X. birchmanni* ancestry on chromosome 7 or 14 is only under selection in combination with another nuclearly encoded locus, this approach will underestimate the strength of selection on such an incompatibility.

### Dissections of pregnant females

We collected pregnant females for two purposes: (1) to conduct paired mother/embryo sequencing to quantify rates of assortative mating in the Chapulhuacanito hybrid population and (2) to evaluate evidence for links between developmental phenotypes and particular genotypes in the F_2_ embryos of F_1_ hybrid mothers. All females were euthanized with an overdose of MS-222. For the Chapulhuacanito hybrid population, each female (*n* = 49) was dissected and the whole ovary containing developing embryos was removed. Embryos were examined under a dissection scope to determine if they had been fertilized (that is, evidence of a forming blastodisc or morphological evidence of later developmental stages^52^). Embryos were visually inspected for any developmental delay or asynchrony, which has been linked to hybrid incompatibilities in previous work^35^. At least two embryos were randomly selected for DNA extraction (*n* = 101) and sequencing from each female and a fin clip was taken from the female. For the F_1_ females (*n* = 4), we selectively identified individuals with expanded gravid spots, suggestive of these individuals being in the late stages of pregnancy. Embryos were dissected out of the ovary and developmentally staged following the same procedure as for Chapulhuacanito females (*n* = 126 embryos) and broods were additionally photographed under a dissection scope. All embryos underwent DNA extraction and samples were prepared for sequencing as described above.

### Female mate preference assays

We tested female *X. birchmanni* (from the Río Garces 20° 56′ 24.96″ N and 98° 16′ 52.21″ W and the Río Xiliatl 21° 6′ 19.00″ N and 98° 33′ 47.70″ W) and *X. cortezi* (from the arroyo La Conchita 21° 20′ 6″ N and 98° 35′ 35.52″ W) from allopatric populations for their preference for conspecific or heterospecific males in two sets of preference experiments conducted in 2004, 2005 and 2007. Trials were conducted in a 208-l tank divided into five equal sections with two outer sections separated from the inner three with partitions—either (1) solid glass for trials with only visual cues or (2) Plexiglass with 0.25-inch diameter holes every 6 inches^2^ for trials with visual and olfactory cues. Fish were allowed to acclimate for 10 min before trials began: one male from each species was placed in either of the two outer sections of the tank and a female was placed in the centre in a clear holding cube. We released females and recorded the time they spent in the inner sections adjacent to each male through a window covered with one-way glass for a 10-min period. To control for any side bias, we then switched the placement of the two males and repeated the experiment. We repeated these trials with the same trio of individuals after a 7-day period (for a total of four trials for each female; two separate ‘pairs’ for analysis). Males were paired in these trials to minimize size differences as much as possible (mean absolute size difference; visual trials, 1.01 ± 0.23 mm; visual with olfactory trials, 0.6 ± 0.1 mm). For trials with only visual cues, we tested 21 *X. birchmanni* and 10 *X. cortezi* females. For trials with visual and olfactory cues, we tested 19 *X. birchmanni* and 18 *X. cortezi* females. The trial setup did not provide females the ability to detect ultraviolet cues on the males, although we do not expect this to impact female preference, as the closely related *X. malinche* reflects minimally in the ultraviolet^57^. Behavioural experiments were approved by the Animal Care Guidelines of Ohio University (Animal Care and Use approval no. L01-01) and Stanford Laboratory Animal Care (protocol no. 33071).

To account for side bias, we removed females from our analyses if they spent >80% of their time on one side of the tank during an experiment. This resulted in 41 retained *X. birchmanni* and 18 retained *X. cortezi* experiments for the visual trials and 37 retained *X. birchmanni* and 30 retained *X. cortezi* experiments for the visual with olfactory trials. Time spent associating with males is correlated with female mating decisions in *Xiphophorus*^58,59^, so we calculated the time spent with *X. birchmanni* and *X. cortezi* males in each pair of trials to calculate the strength of preference^60^: the difference between time spent with the *X. cortezi* male and time spent with the *X. birchmanni* male, divided by the total time spent with either male. The strength of preference varies from +1.0 to −1.0 with positive values indicating a preference for *X. cortezi* males and negative values indicating a preference for *X. birchmanni* males. We used one-sided Wilcoxon signed-rank tests to assess the difference from a null expectation of a strength of preference of 0 (no preference).

## Extended Data

## Supplementary Material

supplementary

supplementary material

## Data Availability

All newly collected DNA sequence data generated for this project are available through the NCBI BioProject PRJNA1106506. Additional data are available on Dryad at https://doi.org/10.5061/dryad.dbrv15f8v (ref. 61).
