# Supplementary material for: Pervasive gene flow despite strong and varied reproductive barriers in swordtails
Source: Nat Ecol Evol. 2025 Mar 26;9(5):867–78. doi: 10.1038/s41559-025-02669-9 (PMC12066355; doi:10.1038/s41559-025-02669-9)
Supplement: Supplementary file 1 [file 41559_2025_2669_MOESM2_ESM.pdf]

nature portfolio

Corresponding author(s): Stefanie M. Aguillon

Last updated by author(s): 01/24/2025

Reporting Summary

Nature Portfolio wishes to improve the reproducibility of the work that we publish. This form provides structure for consistency and transparency in reporting. For further information on Nature Portfolio policies, see our Editorial Policies and the Editorial Policy Checklist.

Statistics

For all statistical analyses, confirm that the following items are present in the figure legend, table legend, main text, or Methods section.

n/a	Confirmed	
☐	☒	The exact sample size (*n*) for each experimental group/condition, given as a discrete number and unit of measurement
☐	☒	A statement on whether measurements were taken from distinct samples or whether the same sample was measured repeatedly
☐	☒	The statistical test(s) used AND whether they are one- or two-sided<br>*Only common tests should be described solely by name; describe more complex techniques in the Methods section.*
☐	☒	A description of all covariates tested
☐	☒	A description of any assumptions or corrections, such as tests of normality and adjustment for multiple comparisons
☐	☒	A full description of the statistical parameters including central tendency (e.g. means) or other basic estimates (e.g. regression coefficient) AND variation (e.g. standard deviation) or associated estimates of uncertainty (e.g. confidence intervals)
☐	☒	For null hypothesis testing, the test statistic (e.g. *F*, *t*, *r*) with confidence intervals, effect sizes, degrees of freedom and *P* value noted<br>*Give P values as exact values whenever suitable.*
☐	☒	For Bayesian analysis, information on the choice of priors and Markov chain Monte Carlo settings
☒	☐	For hierarchical and complex designs, identification of the appropriate level for tests and full reporting of outcomes
☒	☐	Estimates of effect sizes (e.g. Cohen's *d*, Pearson's *r*), indicating how they were calculated

Our web collection on statistics for biologists contains articles on many of the points above.

Software and code

Policy information about availability of computer code

Data collection	Simulated data were collected using SLiM v3.3.1, admix'em v1.0.2.3, and custom R v4.2.1 code available on GitHub at https://github.com/stepfanie-aguillon/swordtail-reproductive-barriers
Data analysis	All code to replicate analyses is available on GitHub at https://github.com/stepfanie-aguillon/swordtail-reproductive-barriers or on Zenodo (https://doi.org/10.5281/zenodo.14736260). Raw sequencing data was processed using local ancestry inference using ancestryinfer (https://github.com/Schumerlab/ancestryinfer) and other custom scripts available on the Schumer Lab GitHub (https://github.com/Schumerlab/Lab_shared_scripts).

For manuscripts utilizing custom algorithms or software that are central to the research but not yet described in published literature, software must be made available to editors and reviewers. We strongly encourage code deposition in a community repository (e.g. GitHub). See the Nature Portfolio guidelines for submitting code & software for further information.

Data

Policy information about availability of data

All manuscripts must include a data availability statement. This statement should provide the following information, where applicable:

- Accession codes, unique identifiers, or web links for publicly available datasets
- A description of any restrictions on data availability
- For clinical datasets or third party data, please ensure that the statement adheres to our policy

Datasets are available on the Dryad Digital Repository (https://doi.org/10.5061/dryad.dbrv15f8v).

All newly collected DNA sequence data generated for this project are available through the NCBI BioProject PRJNA1106506. The phylogenetic analyses additionally use the X. birchmanni reference genome available in the NCBI BioProject PRJNA1043674 (GCA_036418095.1).

Research involving human participants, their data, or biological material

Policy information about studies with human participants or human data. See also policy information about sex, gender (identity/presentation), and sexual orientation and race, ethnicity and racism.

Reporting on sex and gender	NA
Reporting on race, ethnicity, or other socially relevant groupings	NA
Population characteristics	NA
Recruitment	NA
Ethics oversight	NA

Note that full information on the approval of the study protocol must also be provided in the manuscript.

Field-specific reporting

Please select the one below that is the best fit for your research. If you are not sure, read the appropriate sections before making your selection.

☐ Life sciences ☐ Behavioural & social sciences ☒ Ecological, evolutionary & environmental sciences

For a reference copy of the document with all sections, see nature.com/documents/nr-reporting-summary-flat.pdf

Ecological, evolutionary & environmental sciences study design

All studies must disclose on these points even when the disclosure is negative.

Study description	This study tested a series of reproductive barriers between the hybridizing fish Xiphophorus birchmanni and Xiphophorus cortezi using a combination of genomic sequencing from natural hybrid populations, artificial crosses in the laboratory, behavioral assays, sperm performance, and developmental studies.
Research sample	Our study included sampling from a naturally hybridizing population (Chapulhuacanito) of Xiphophorus birchmanni and Xiphophorus cortezi (n = 306) from 2021-2022, as well as historic sampling from this same site (n = 11 in 2003, n = 21 in 2006, and n = 41 in 2017). These samples were adults and juveniles from both sexes. To assess assortative mating within Chapulhuacanito, we sampled 49 pregnant females for embryo dissection and sequencing, resulting in 126 sequenced embryos. We additionally performed behavioral preference assays on females from allopatric populations using live male cues of these two species (visual trials: n = 21 X. birchmanni and n = 10 X. cortezi; visual+olfactory trials: n = 19 X. birchmanni and n = 18 X. cortezi) and from Chapulhuacanito (n = 14 admixed cluster, n = 15 X. birchmanni cluster) and allopatric populations using isolated male olfactory cues (n = 17 X. cortezi, n = 16 X. birchmanni). Assessments of sperm morphology and motility were conducted on 4 laboratory born and reared males from each of the following groups: X. birchmanni, X. cortezi, and their F1 and F2 hybrids. We assessed hybrid inviability by producing F1 hybrids in the laboratory (tracking n = 39 total). Phylogenetic analyses utilized high-coverage sequencing from 35 samples from X. birchmanni, X. malinche, X. cortezi, X. montezumae, X. multilineatus, X. nigrensis, X. pygmaeus, X. nezahualcoyotol, X. clemenciae, and X. helleri. To assess mitonuclear incompatibilities, we produced X. cortezi x X. birchmanni F2 hybrids in the laboratory. We first assessed 127 F2 embryos dissected from 4 pregnant females. We then leveraged 163 F2s that survived to adulthood.
Sampling strategy	Sample sizes for analyses using individuals collected from natural populations were determined by sampling success during a specific field excursion and the limits across all sites issued by the collecting permits. Sample sizes for analyses using lab-born individuals were the total number available for genotyping or the maximum feasible while maintaining viability and health of the lab colonies.
Data collection	Detailed information on data collection are included in the main text and supplement. QKL, JJB, SMB, and AED performed molecular

Data collection	lab work for sequencing. SMA, QKL, and MS performed bioinformatics. SMA and BMM collected data on wild-caught and lab-produced embryos. TRG, SMA, and DLP led animal husbandry to produce lab-generated F1 and F2 hybrids. SMA, SKHC, CG-R, OR-C, and MRM performed behavioral preference assays. DLP assessed sperm morphology and collected sperm motility data. MS constructed phylogenies. PF-Z collected and processed video observations. SMA and MS performed all simulations and statistical analyses.
Timing and spatial scale	Fieldwork was primarily conducted in the Río San Pedro river drainage of San Luis Potosí, México in 2021-2022 at the Chapulhuacanito sampling site. Additional historical population samples from the Río San Pedro were collected in 2003, 2006, and 2017. Behavioral assays using live male cues were conducted in 2004, 2005, and 2007 using fish collected from the Río Garces, the Río Xiliatl, and the arroyo La Conchita (all in Hidalgo, México).
Data exclusions	Samples were excluded from the Chapulhuacanito population sampling if sequencing did not result in at least 300,000 raw sequencing reads. Poor sequencing quality can be a result of issues in the laboratory or because the tissue sample obtained in the field was too small. This cut-off was established before conducting any downstream analyses based on prior work in the system.
Reproducibility	Large datasets collected over multiple years/collection trips were used to ensure reproducibility of results for the population sampling at Chapulhuacanito. For behavioral preference assays with relatively small sample sizes, we conducted analyses to assess our statistical power (finding that we had good power to detect strong preferences with our sample size, but were underpowered to detect weaker preferences). We additionally conducted power analyses for the detection of loci with a range of selection coefficients in our sample of adult F2 hybrids. Power only exceeded 50% at selection coefficients of 0.5 and exceeded 80% at selection coefficients of 0.6, and we thus expect that there are many loci under selection in F2 hybrids that we are failing to detect here.
Randomization	We did not perform experiments in this study that required randomization.
Blinding	Blinding occurred only during the experimental behavioral assays of Chapulhuacanito females where differences in genotypic clusters were of interest. We performed all behavioral trials blind to female genotype (presence in the X. birchmanni or admixed ancestry cluster) and subsequently matched females to their genotypes using sequencing.

Did the study involve field work? ☒ Yes ☐ No

Field work, collection and transport

Field conditions	Fieldwork was primarily conducted in the Río San Pedro river drainage of San Luis Potosí, México in 2021-2022. Additional historical population samples from the Río San Pedro were collected in 2003, 2006, and 2017. Behavioral assays were conducted in 2004, 2005, and 2007 using fish collected from the Río Garces, the Río Xiliatl, and the arroyo La Conchita (all in Hidalgo, México). Environmental conditions are variable in this region of México depending on the time of year: with temperatures ranging from 10-30C depending on the season and rainfall ranging from absent (peak dry season in winter/spring) to substantial (peak rainy season in summer/fall).
Location	The primary sampling site for this work was the Chapulhuacanito population (21°12'10.58"N 98°40'28.27"W), but fish from the allopatric populations of Puente de Huichihuayán (21°26'9.95"N 98°56'0.00"W) and Coacuilco (21°5'51.16"N 98°35'20.10"W) were used to produce artificial crosses in the laboratory, and fish from the Río Garces (20°56'24.96"N 98°16'52.21"W), Río Xiliatl (21° 6'19.00"N 98°33'47.70"W), and arroyo La Conchita (21°20'6"N 98°35'35.52"W) were also used in behavioral assays. Sampling was conducted in less than 2 meters of water at all sites.
Access & import/export	Samples were collected with permission from the Mexican National Commission of Aquaculture and Fisheries (Permit No. PPF/ DGOPA-002/19) and imported under Long-Term Importation Permit #2023 - 7129 from the California Department of Fish and Game.
Disturbance	Baited minnow traps are used for sample collection and all non-target species are released back into their original pool. Fish that are fin clipped are allowed to recover in river water before being released back into their original pool. No disturbance was caused by the study other than removal of some Xiphophorus individuals from the streams (either temporarily for fish that were fin clipped or permanently for the small number of fish euthanized after collection). Populations are frequently monitored for signs of disturbance caused by sampling (e.g., changes in sex or age distribution).

Reporting for specific materials, systems and methods

We require information from authors about some types of materials, experimental systems and methods used in many studies. Here, indicate whether each material, system or method listed is relevant to your study. If you are not sure if a list item applies to your research, read the appropriate section before selecting a response.

Materials & experimental systems

n/a	Involved in the study
☒	☐ Antibodies
☒	☐ Eukaryotic cell lines
☒	☐ Palaeontology and archaeology
☐	☒ Animals and other organisms
☒	☐ Clinical data
☒	☐ Dual use research of concern
☒	☐ Plants

Methods

n/a	Involved in the study
☒	☐ ChIP-seq
☒	☐ Flow cytometry
☒	☐ MRI-based neuroimaging

Animals and other research organisms

Policy information about studies involving animals; ARRIVE guidelines recommended for reporting animal research, and Sex and Gender in Research

Laboratory animals: We utilized wild-caught and laboratory born fish in this study. Including male and female Xiphophorus birchmanni, X. cortezi, their natural hybrids, and their lab-born F1 and F2 hybrids.

Wild animals: Natural hybrids between X. birchmanni and X. cortezi were collected from the Chapulhuacanito population using baited minnow traps (n = 306). After anesthetization, a small fin clip was taken from the upper caudal fin of each individual and preserved in 95% ethanol for DNA extraction. Fish were allowed to recover from anesthetization in river water before being released at their collection site. A subset of pregnant females from Chapulhuacanito (n = 49) were euthanized in an overdose of MS-222 and preserved in 95% ethanol for paired mother/embryo sequencing. We also took advantage of historical samples from 2003 (n = 11), 2006 (n = 21), and 2017 (n = 41) at Chapulhuacanito collected through a companion study. These samples were preserved in either DMSO or 95% ethanol at the time of collection. Individuals were transported in minnow buckets for short term travel and in containers of methylene-blue treated water for importation.

Reporting on sex: Sex was not considered in the majority of analyses included in this study. The exceptions include: assessment of female mating preferences through behavioral assays and the assessment of sperm morphology and motility differences in males.

Field-collected samples: Differences in lab rearing condition (e.g., housing, temperature, photoperiod, or general maintenance) were not used as experimental conditions in this study.

Ethics oversight: Animal work was approved by the Animal Care Guidelines of Ohio University (Animal Care and Use approval no. L01-01) and Stanford Laboratory Animal Care (protocol no. 33071)

Note that full information on the approval of the study protocol must also be provided in the manuscript.

Plants

Seed stocks: NA

Novel plant genotypes: NA

Authentication: NA
